# Virtual Reality and Gamification for Assessing Technical Aptitude, Cognitive Abilities, and Personality Characteristics in Surgical Residency Selection: Validation Study

**DOI:** 10.2196/82515

**Published:** 2026-05-25

**Authors:** Noa Gazit, Gilad Ben-Gal, Ron Eliashar

**Affiliations:** 1 Department of Otolaryngology/HNS, Faculty of Medicine, Hebrew University of Jerusalem, Hadassah Medical Center, Kalman Ya'akov Man 1, Jerusalem, 9112001, Israel, 972 547567448; 2 Department of Prosthodontics, Faculty of Dental Medicine, Hebrew University of Jerusalem, Hadassah Medical Center, Jerusalem, Israel

**Keywords:** resident selection, assessment, surgical training, technical aptitude, cognitive abilities, personality characteristics, surgical simulators, gamification, game-based assessment

## Abstract

**Background:**

Assessment of technical aptitude, cognitive abilities, and personality characteristics is important in selecting candidates for surgical training. Currently, the selection of surgical training candidates does not systematically include objective assessment of these variables. Instead, it relies heavily on traditional selection methods, such as academic achievement, letters of recommendation, and interviews, whose presumed relationships with later performance are based on limited and inconsistent evidence.

**Objective:**

This study examined evidence for validity based on relationships with other variables—a key source of validity evidence—to support the use of 2 novel tools for objectively assessing surgical training candidates: a nonimmersive (screen-based) virtual reality laparoscopic technical aptitude test, and a game-based assessment of cognitive abilities and personality characteristics.

**Methods:**

This study had 3 phases, all focused on establishing validity based on relationships with other variables. In Phase 1, we evaluated convergent and discriminant evidence of validity by assessing the correlation between interns’ performance in the 2 novel selection tests and in 4 established psychometric instruments for assessing dexterity, visuospatial ability, intelligence, and personality. In Phase 2, we evaluated evidence for the test-criterion relationship by assessing the correlation between residents’ performance in the 2 novel tests and their concurrent residency performance evaluations. In this phase, we also assessed evidence for the fairness of the tests between genders. In Phase 3, we focused on the technical aptitude test and evaluated evidence for its relationship with training level by administering the test to a sample of expert surgeons and comparing their performance with that of the residents and interns from the previous phases.

**Results:**

Interns’ scores on the 2 novel selection tests were correlated with scores on the relevant established psychometric instruments, providing convergent and discriminant evidence (Phase 1). Residents’ scores on the 2 novel tests were significantly correlated with relevant performance criteria (Phase 2). In addition, no evidence for gender bias in the tests was found. Finally, based on data collected in all 3 phases, we found evidence for expert-novice differences in the technical aptitude test scores, such that scores were correlated with surgical experience.

**Conclusions:**

The findings provide validity evidence supporting the use of the novel virtual reality–based technical aptitude test and a game-based assessment of cognitive abilities and personality characteristics in selecting candidates for surgical training. However, because the test-criterion evidence was obtained using a concurrent design, further prospective longitudinal studies are required to determine whether these assessments predict subsequent residency performance.

## Introduction

### Background

The selection of candidates for surgical training is a crucial step in ensuring the quality and safety of patient care. The primary objective of the selection process is to identify the most suitable candidates, those who will perform well both during their residency and ultimately as independent surgeons. For an effective selection process for surgical training, it is widely accepted that prospective candidates should be assessed objectively on their technical aptitude (eg, dexterity, coordination, and visuospatial ability), cognitive abilities (eg, deductive and inductive reasoning, learning ability, and concentration), and personality characteristics (eg, decision-making, stress tolerance, and communication skills) [1-7].

In practice, many surgical training programs have formalized and structured selection procedures. However, these procedures rely predominantly on traditional selection methods such as academic achievement, letters of recommendation, and interviews, which do not systematically incorporate standardized, performance-based assessments [8,9]. Evidence regarding the relationships between these traditional methods and subsequent clinical and operative performance during residency is mixed and context-dependent, with several studies demonstrating only weak to modest associations [10-14]. Efforts to identify more suitable tools for objectively assessing candidates’ capacities and characteristics have included surrogate tests for assessing technical aptitude (indirect indicators of nonspecific technical abilities, usually in paper-and-pencil or computerized formats); medical exams (eg, the USMLE [United States Medical Licensing Examination]) for assessing cognitive abilities; and self-report questionnaires for assessing personality characteristics (eg, the “Big Five” personality traits, emotional intelligence, and grit). However, there is as yet no consistent evidence that these methods improve the selection of surgical residents [4,10,15-17]. The limited and inconsistent validity evidence supporting these approaches may partly explain why their use has remained largely confined to experimental or pilot contexts, with limited translation into routine, large-scale selection practice.

This research examines an alternative approach to the selection of surgical residents based on innovative simulation assessment methods [18,19], similar to those used in the selection process for aircrew [20]. Through simulation, examinees are exposed to controlled situations designed to elicit behaviors relevant to the assessment of particular competencies. These situations can be designed so that they mimic the challenges and tasks associated with real-life surgical work. Therefore, these methods are expected to be more effective than traditional methods of assessment or other surrogates tried thus far.

Simulative testing can be conducted either in the real world by evaluators or actors, or on a computer or a simulator, using innovative methods such as virtual reality (VR) and gamification. VR is broadly defined as a computer-generated simulation of a realistic 3D environment in which users interact with digitally constructed stimuli in real time [21]. VR systems are characterized by varying degrees of presence (the subjective sense of “being there”), immersion (the extent to which the system technologically replaces or augments sensory input), and interactivity (the user’s ability to influence the virtual environment through active responses) [22]. According to established classifications, VR technologies range from nonimmersive desktop-based systems displayed on standard monitors to fully immersive head-mounted display environments. Nonimmersive systems, while providing lower levels of sensory input, nonetheless allow real-time interaction with a computer-generated environment and performance-based feedback.

Gamification refers to the incorporation of game design elements, such as goals, rules, feedback systems, scoring mechanisms, levels, and reward structures, into nongame contexts in order to enhance engagement, motivation, and behavioral activation [23]. This approach has led to the development of game-based assessments (GBAs), which embed psychometrically informed measurement principles within interactive digital environments [24-27]. GBAs emerged within educational technology as part of a broader shift toward digital and data-driven assessment, alongside efforts to enhance learning through adaptive personalization [28]. Rather than relying solely on final test scores, such assessment approaches analyze in-game behavioral data, such as response times, movement trajectories, action sequences, error patterns, decision latency, and changes in strategy following feedback, to infer underlying cognitive and noncognitive competencies [29]. Advances in computational psychometrics then allow modeling these behavioral traces using principled statistical frameworks in order to link observable digital actions to latent constructs, such as problem-solving, working memory, persistence, visuospatial reasoning, flexibility, or decision-making under uncertainty [30]. In parallel, evidence-centered design frameworks provide structured approaches for aligning task design, observed behaviors, and intended score interpretations within immersive digital environments [31]. In the domain of personnel selection, GBAs improve on traditional self-report questionnaires or decontextualized cognitive tests because they allow the inference of job-relevant skills, abilities, and personality characteristics from observable gameplay behaviors, including response times, decision patterns, error rates, learning curves, and adjustments in strategy following feedback or changing task demands. These behavioral traces are captured continuously and transformed into quantifiable performance indicators, as described above.

Importantly, the VR and gamification paradigms are not mutually exclusive. In particular, a simulation-based assessment may use VR technology while simultaneously incorporating gamified design elements such as structured goals, scoring systems, feedback mechanisms, and progressive task levels. This study focuses on the validation of 2 separate tools: a game-based assessment administered on a standard computer for measuring cognitive abilities and personality characteristics; and a screen-based laparoscopic virtual reality simulator for assessing technical aptitude. However, the VR-based test also incorporates several gamified elements intended to structure performance and enhance engagement (see the description of the tests in the “Methods” section for more details). Both tools are designed to support standardized measurement and defensible score interpretation in a high-stakes surgical selection context.

VR and gamification are promising directions in assessment that have numerous advantages over conventional methods [24-26]. First, they promote a more positive assessment experience that reduces examinees’ stress levels and increases their engagement and motivation. Second, the use of computerized systems allows collecting rich, high-resolution spatiotemporal behavioral data, which provides a great deal of information about each participant’s performance [32,33]. Since performance is assessed continuously throughout the tasks based on multiple parameters, this results in more valid and reliable assessments. Finally, these assessments are based on an automated scoring system, which eliminates the bias associated with human assessments [34,35] or self-report questionnaires [36,37]. These features of VR-based simulation and game-based assessment may contribute to more valid and reliable evaluation of candidates’ skills and abilities.

The implementation of VR and gamification in assessment is a relatively new direction in personnel selection in general, and in the selection of candidates for surgical training in particular. Although a few studies have provided initial evidence regarding the potential of using VR simulators to assess candidates’ technical aptitude [38-42], there is not yet sufficient evidence supporting the validity of these simulators for selecting candidates for surgical training [43] (evidence for test content, response process, internal structure, relationships with other variables, and consequences, as described by Messick [44,45]). In addition, studies exploring the implementation of VR or gamification for assessing the cognitive abilities and personality characteristics of candidates for medical residency programs (surgical or nonsurgical) are scarce.

### Study Objectives

This paper is part of a larger research project addressing the systematic development and validation of 2 new simulation-based assessments for resident selection which integrate novel VR and gamification techniques [46,47]: a technical aptitude test performed on the nonimmersive (screen-based) Lap-X-VR laparoscopic simulator [48]; and a computerized GBA of cognitive abilities and personality characteristics, which comprises 3 video games designed specifically for assessment.

Consistent with contemporary validity theory, validity is not a property of a test itself but of the interpretation and proposed use of its scores [49]. According to Messick’s unified framework, validity is supported by evidence from multiple sources, including content, response processes, internal structure, relationships with other variables, and consequences [44,45]. In 2 previous studies [46,47], we presented initial evidence primarily addressing content, response process, and internal structure to support the proposed interpretation and use of these tests in surgical residency selection. The aim of this study was to extend this validation program by examining evidence based on relationships with other variables—that is, the degree to which the relationships of the test scores with other variables are consistent with the construct underlying the proposed interpretation of the scores [44,45].

Specifically, this study addressed the following research questions:

1. Do scores on the VR-based technical aptitude test and the GBA demonstrate convergent and discriminant associations with established psychometric measures of related and unrelated constructs?
2. Are test scores associated with residency performance evaluations, thereby providing evidence of test-criterion relationships?
3. Do VR technical aptitude scores differentiate between interns, residents, and expert surgeons (expert-novice differences)?
4. Given previously observed gender differences in test scores, is there evidence of differential prediction with respect to gender?

To address these questions, we conducted a multiphase validation study involving interns, residents, and expert surgeons, using correlational and group comparison analyses within a validity framework. We hypothesized that (1) both assessments would exhibit construct-consistent patterns of convergent and discriminant associations, with stronger relationships between each test and theoretically aligned constructs than with unrelated measures, (2) scores on both assessments would be positively associated with relevant dimensions of residency performance evaluations, (3) scores on the VR technical aptitude test would increase with surgical experience, and (4) although mean gender differences might be observed, the tests would not demonstrate evidence of differential prediction.

The findings are intended to inform stakeholders responsible for the selection of candidates for surgical training, including program directors, medical educators, and researchers seeking evidence-based tools for high-stakes decision-making.

## Methods

### Overview of the Validation Study Design

This study had 3 main phases, each designed primarily to collect evidence for one set or type of relationships with other variables: relationships with other tests measuring similar and different constructs (convergent and discriminant evidence), test-criterion relationships, and expert-novice differences [44,45,50]. Additional analyses, including incremental evidence of validity and fairness (differential prediction), were conducted within this overarching framework of relationships with other variables. Table 1 provides an overview of the validity evidence collected and the analytic approaches used to evaluate each source.

**Table 1.** Summary of evidence collected to evaluate validity (relationships with other variables) and fairness.

Type of evidence	Definition	Relevant study phases^a^	Evidence collected in the study
Convergent and discriminant evidence	Evidence based on assessing the relationship between the selection tests and other instruments. Convergent evidence for validity refers to the relationships between test scores and other measures intended to assess the same or similar constructs, while discriminant evidence for validity refers to the relationships between test scores and other measures intended to assess different constructs	Phase 1	Associations between the novel selection tests and 4 established measures of dexterity and coordination, visuospatial ability, intelligence, and personality^b^. The relationship between the VR^c^-based technical aptitude test and both dexterity and coordination and visuospatial ability (the PPT^d^ and MRT^e^), and between the GBA^f^ and both intelligence and personality (the RAPM^g^ and the mini-IPIP^h^), was considered convergent evidence; the relationship between the VR-based technical aptitude test and intelligence and personality, and between the GBA and dexterity and coordination and visuospatial ability, was considered discriminant evidence.
Test-criterion relationships	Evidence based on assessing the relationship between the selection tests and relevant external performance criteria, reflecting the extent to which test scores meaningfully predict measures of real-world performance	Phase 2	Associations between the novel selection tests and 16 residency performance criteria (1 technical skills criterion and 15 nontechnical skills criteria) were examined in a concurrent design. The criteria were assessed using structured evaluations completed independently by 3 to 4 supervising surgeons per resident. The technical skills criterion was used to evaluate the VR-based technical aptitude test, while the 15 nontechnical criteria were used to evaluate the GBA.
Incremental evidence	Evidence based on examining whether a test explains unique variance in a relevant outcome beyond that accounted for by other measures, thereby supporting its incremental contribution within the assessment framework	Phase 2	Associations were examined to determine whether each selection test predicted its theoretically relevant performance criterion above and beyond the other test. The incremental contribution of the VR-based technical aptitude test was evaluated for the technical skills criterion beyond the GBA, and the incremental contribution of the GBA was evaluated for the aggregated nontechnical performance criteria beyond the VR-based technical aptitude test.
Relationship with training level (expert-novice differences)	Evidence based on examining the relationship between selection test scores and theoretically relevant variables (eg, training level for the technical aptitude test), reflecting the extent to which these associations are consistent with the construct underlying the proposed interpretation of the scores	All phases	Performance on the VR-based technical aptitude test was compared across interns, residents, and expert surgeons to evaluate whether scores increased with level of surgical expertise. Expert-novice differences were not evaluated for the GBA.
Fairness (differential prediction)	Evidence based on examining whether test scores predict relevant performance criteria equivalently across demographic groups (eg, gender)	Phase 2	For each selection test, regression models were estimated to determine whether gender significantly moderated the relationship between test scores and the relevant performance criterion (technical skills for the VR-based technical aptitude test and the aggregated nontechnical performance criteria for the GBA). Sensitivity analyses were conducted, including prior simulator experience and prior video game experience as covariates, to assess the robustness of the findings.

a The study included 3 phases: Phase 1 (administration to interns), Phase 2 (administration to residents), and Phase 3 (administration to expert surgeons).

b For details on these tests and their sources, see under “Phase 1: Administration to Interns,” below, and Table S1 in the Multimedia Appendix 1.

c VR: virtual reality.

d PPT: Purdue Pegboard Test.

e MRT: Mental Rotation Test.

f GBA: game-based assessment.

g RAPM: Raven Advanced Progressive Matrices.

h mini-IPIP: short version of the International Personality Item Pool.

In Phase 1, the 2 novel selection tests (ie, both the VR technical aptitude test and the GBA of cognitive abilities and personality characteristics) were administered to a sample of interns, along with 4 established psychometric instruments commonly used for assessing dexterity and coordination, visuospatial ability, intelligence, and personality. Based on the data collected in this phase, we evaluated evidence for the relationships between scores on the 2 novel selection tests and other tests measuring similar and different constructs (convergent and discriminant evidence). In Phase 2, the 2 novel tests were administered to a sample of residents, and their performance in residency was assessed using an evaluation form filled in by their supervisors in a concurrent design (obtaining test scores and criterion information simultaneously). This phase allowed us to assess evidence for test-criterion relationships. In addition, the data from this phase were used to assess the incremental contribution of each test and evaluate evidence for the possibility of gender bias in the tests. Finally, in Phase 3, the VR technical aptitude test was administered to a sample of senior surgeons. Their scores were then compared with those of the interns and residents from the previous phases in order to test for expert-novice differences (ie, the relationship between the technical aptitude scores and test-takers’ training level). Expert-novice differences were assessed only for the VR technical aptitude test and not for the GBA of cognitive abilities and personality characteristics because evidence regarding expert-novice differences is relevant only when the construct being measured is hypothesized to be related to training status and, therefore, should differ between the groups [49,51,52]. Unlike the technical aptitude test, the tasks used in the GBA do not resemble real-life surgical tasks or challenges. Therefore, expert-novice differences are not relevant to the GBA.

Formal a priori power calculations to determine sample sizes for the 3 phases were not performed due to the feasibility-based recruitment framework and the fixed pool of eligible participants. Based on practical and logistical considerations, we aimed to recruit a minimum of 60 participants per training level while including all available individuals. Given the final sample (n=216), the study was adequately powered to detect small-medium effect sizes for between-group comparisons, within-group correlations, and regression analyses.

### Ethical Considerations

This study was approved by the ethics committee of the Hebrew University of Jerusalem (approval no. 13032023) and was conducted in accordance with the principles of the Declaration of Helsinki. All participants provided informed consent prior to participation. Participant data were stored using unique anonymized identifiers; the key linking these identifiers to real identities was kept in a password-protected file stored offline, ensuring that no identifying information was accessible online. Interns received US $70 and residents US $50 as monetary compensation. Both groups were also provided with feedback on their performance in the test, presented as percentile rankings relative to their respective samples.

### The Selection Tests

This section briefly describes the 2 novel tests examined in this research.

#### The VR Technical Aptitude Test

**Figure 1.**
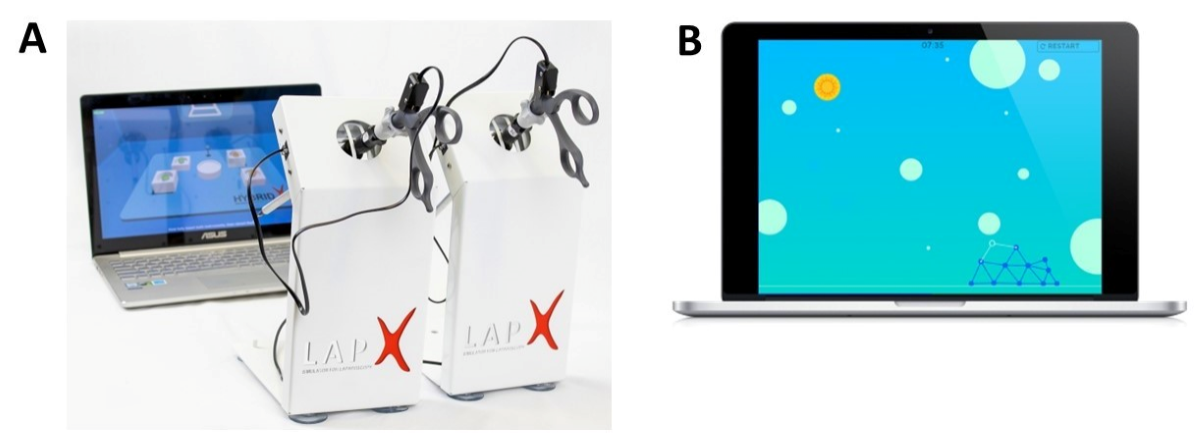
Illustrations of the 2 selection tests evaluated in the study. (A) The technical aptitude test was performed on the LAP-X-VR laparoscopic simulator. (B) The game-based assessment of cognitive abilities and personality characteristics. For illustration, only one task of each test is presented.

The VR technical aptitude test was developed and designed to assess technical aptitude among candidates for surgical training with no former surgical experience or knowledge [46]. The test consists of 10 tasks designed to assess the psychomotor and perceptual abilities (coordination, ambidexterity, movement precision, visuospatial ability, and depth perception) needed to perform key tasks relevant to minimally invasive surgery (MIS), including grasping and transferring objects, cutting with scissors, scope handling, and suturing with a needle [40,53]. The test is performed on the Lap-X-VR laparoscopic simulator (Medical-X; Figure 1A) [48] and takes about 50 minutes to complete. The Lap-X-VR is a nonimmersive, screen-based laparoscopic VR simulator. Examinees manipulate real laparoscopic instruments connected to a computer system displaying a 3D virtual operative field on a standard monitor. Unlike fully immersive VR systems, it does not use head-mounted displays, stereoscopic visualization, or spatial head tracking. The simulated operative field is displayed on a conventional monitor, and immersion is therefore limited to a screen-based interaction.

Although primarily designed as a VR-based surgical simulator, the test also incorporates several gamified elements intended to structure performance and enhance engagement, including clearly defined task goals, real-time performance feedback, scoring metrics, and progressive task completion requirements. As such, it reflects the integration of VR technology and gamification principles within a single assessment platform.

Test scores are calculated based on the aggregation of performance data recorded by the simulator in each task for the following parameters: success rate (%), time (s), number of mistakes, path length (cm), and, where relevant, percent of time within scope (%). Final test scores are presented on a scale with a mean of 100 and an SD of 20, with higher scores indicating better technical aptitude. This test has been shown to have high reliability (Cronbach α=0.83) and discrimination between examinees (mean task discrimination 0.5, SD 0.1) [46]. For a full description of the test development and the specific tasks included, refer to Gazit et al [46].

#### GBA of Cognitive Abilities and Personality Characteristics

The GBA test [47] was developed and designed to assess 14 cognitive abilities and personality characteristics relevant to surgical training [7]: planning, problem-solving, ingenuity, goal orientation, self-reflection, endurance, analytical thinking, learning ability, flexibility, concentration, conformity, multitasking, working memory, and precision. The test was developed in cooperation with Benchmark.games Ltd, a company that produces GBAs for organizational hiring and recruitment. The test is administered on a standard computer and takes about 60 minutes to complete (Figure 1B). The test consists of 3 video games, designed by a team of psychometricians and psychologists. Each video game was designed to assess a set of specific competencies relevant to surgical residents. In each game, all actions of examinees (eg, mouse movements and key presses) are recorded and logged. These raw data are then transformed into higher-level variables that describe a set of meaningful measurements (eg, time to first response, time between actions, accuracy, number of steps, and learning curve). Then, competency scores are calculated by aggregating the relevant variables, with higher weight given to variables characterized by larger variance between candidates. Competency scores are presented on a scale of 1‐10. Final test scores are calculated by averaging the 14 individual competency scores. As with the VR technical aptitude test described above, total scores are then presented on a scale with a mean of 100 and an SD of 20. This test has been shown to have high reliability (Cronbach α=0.76) and discrimination between examinees (mean game discrimination 0.4, SD 0.2) [47]. For a comprehensive description of the test development process, the structure and objectives of the 3 games, the specific cognitive and personality competencies assessed, and the theoretical and empirical rationale underlying their design, see Gazit et al [47].

Note that, consistent with established standards for high-stakes assessment [45] and common practice in commercially developed game-based selection tools [24,27], both Gazit et al [47] and this paper report only the tool’s general scoring framework and psychometric properties. Detailed operational definitions of specific behavioral indicators and exact weighting coefficients are proprietary to Benchmark.games Ltd and protected under contractual confidentiality agreements, precluding disclosure of precise scoring parameters. Maintaining confidentiality of specific scoring rules also serves test security purposes in high-stakes contexts. In simulation-based assessments, validity depends partly on candidates responding naturally to task demands rather than optimizing behavior toward explicitly known scoring indicators. Disclosure of detailed indicator-competency mappings could facilitate superficial behavioral adjustments without corresponding changes in the underlying competencies, thereby introducing construct-irrelevant variance.

### Phase 1: Administration to Interns

#### Participants

Seventy-six medical interns from 2 hospitals in Israel participated in Phase 1. To recruit participants, invitations were posted in relevant Facebook (Meta Platforms, Inc) and WhatsApp (WhatsApp LLC) groups along with the contact information of the research coordinator. To improve the likelihood that the sample would represent the population of candidates for surgical training, only interns who were interested in pursuing a surgical career were eligible to participate in the study. Recruitment continued until we had at least 60 participants.

#### Procedure

Each intern was invited to 2 sessions. During the first session, participants completed the 2 novel selection tests. The order in which the tests were administered was randomized across participants. In the second session, participants completed 4 established psychometric instruments measuring dexterity and coordination, visual-spatial ability, intelligence, and personality (see below). Again, the order in which the tests were administered was randomized across participants. The first session lasted approximately 2 hours, and the second about 75 minutes.

The 4 established psychometric instruments were as follows:

1. The Purdue Pegboard Test (PPT) [54,55], a general measure of manual dexterity and bimanual coordination. In the original test, scores are calculated separately for each of the 4 tasks included in the test. In this study, we also calculated total test scores by transforming the 4 raw task scores into z-scores (ie, distributions with a mean of 0 and an SD of 1) and then averaging the z-score values. This was done in order to simplify the interpretation of the relationship between scores on this test and on the selection tests evaluated in this study.
2. The Mental Rotation Test (MRT) [56,57], a general measure of depth perception and visuospatial ability. The original version of this test was developed by Vandenberg and Kuse [56]. In this study, we used a redrawn version of the MRT (the MRT-A) created by Peters et al [57].
3. The Raven’s Advanced Progressive Matrices (RAPM) [58], a nonverbal test used to measure general intelligence and abstract reasoning.
4. The short version of the International Personality Item Pool (mini-IPIP) [59], which measures the personality traits captured in the well-established five-factor model (extraversion, agreeableness, conscientiousness, neuroticism, and openness).

These are all commonly used, well-studied tests that have been validated for assessment of competencies similar to the competencies measured in the 2 novel tests, making them appropriate for evaluating convergent and discriminant evidence of validity. Full descriptions of the 4 tests can be found in Table S1 in the Multimedia Appendix 1.

At the end of the second session, participants provided general demographic information (age, gender, and dominant hand). Participants also reported their previous experience using laparoscopic simulators and playing video games, both on 5-point Likert scales (1=no experience, 5=very extensive experience).

### Phase 2: Administration to Residents

#### Participants

Seventy-five residents from 2 hospitals in Israel participated in Phase 2. To recruit participants, emails and WhatsApp messages were sent to eligible residents asking for their participation. Email addresses and phone numbers of potential participants were obtained from hospital websites or from the database of the Israeli medical association. We contacted only residents in 5 surgical fields characterized by extensive use of MIS techniques: general surgery, gynecology, orthopedics, otorhinolaryngology and head and neck surgery, and urology. Only residents in their second year of residency or above were eligible to participate in the study. We set this exclusion criterion to ensure that participating residents would have sufficient experience in residency that (1) their skills and characteristics would be distinct from those of the interns in Phase 1, and (2) their performance could be reliably evaluated by their supervisors. Recruitment continued until we had at least 60 participants, with at least 5 from each of the 5 surgical fields mentioned above. Participants in this phase had completed on average 3.5 (SD 1.8) years of specialist surgical training.

#### Procedure

Each resident was invited to one 2-hour session. Participants first completed the 2 novel tests. As in Phase 1, the order in which the tests were administered was randomized across participants. They then provided general demographic information (age, gender, dominant hand, and surgical specialty), and reported their previous experience using laparoscopic simulators and playing video games as in Phase 1.

We assessed the residents’ performance in their training program through structured performance evaluations completed by participants’ supervisors (eg, their department directors, training program directors, or other senior expert surgeons who worked closely and directly with the residents). The evaluations assessed residents on 16 dimensions, including the competencies assessed by the 2 novel selection tests (see below). Each resident was evaluated by 3 to 4 supervisors, depending on the specialty. Supervisors filled in all evaluation forms independently, without knowing the ratings of other evaluators, and were blinded to participants’ scores in the 2 selection tests. Participants were informed that we would contact their departments in order to collect their performance evaluations, and we asked them to sign a consent form for this purpose. Four residents declined to sign the form, and so evaluations were used for only 71 of the 75 residents in the sample.

The evaluation form was designed using online survey software (Qualtrics; Qualtrics International Inc) and was sent to supervisors by email. Each supervisor was asked to read an introduction and presentation of the study’s aims before completing the evaluation forms, one for each resident in their department. The evaluations assessed residents’ performance in 16 dimensions: (1) medical knowledge (relative to the resident’s stage in the specific training program), (2) technical skills (again, relative to the resident’s stage in the program), (3) communication with patients and their families, (4) communication with medical staff and teamwork, (5) integrity, (6) diligence, (7) learning ability, (8) decision-making and problem-solving, (9) self-criticism and ability to learn from mistakes, (10) thoroughness, (11) organization and planning, (12) physical and mental endurance, (13) stress tolerance, (14) creativity and cognitive flexibility, (15) motivation, and (16) general assessment of performance in the residency program. Evaluations were provided on a 5-point Likert scale, where 1=very low and 5=very high. Evaluators were required to provide ratings for each of the 16 dimensions.

The evaluation form was developed based on existing forms used in the participating departments to assess residents’ performance and progress. As such, the form included not only competencies assessed by the 2 selection tests examined in this study (eg, technical skills, decision-making and problem-solving, and learning ability), but also competencies not assessed by the 2 selection tests examined here (eg, communication with patients and their families, communication with medical staff and teamwork, and integrity). This decision was made for 2 reasons. First, it allows us to examine whether correlations between the selection tests and competencies that are expected to be related to them are greater than correlations between the selection tests and competencies that are not expected to be related. Second, it enables us to consider residents’ scores on the selection tests in light of a full and comprehensive assessment of their performance in the residency program.

### Phase 3: Administration to Expert Surgeons

#### Participants

Sixty-five senior surgeons from 2 hospitals in Israel participated in Phase 3. To recruit participants, emails and WhatsApp messages were sent to eligible surgeons asking for their participation. Email addresses and phone numbers of potential participants were obtained from hospital websites or from the Israeli Medical Association database. Eligible surgeons were specialists in 1 of the 5 surgical fields from which we drew the resident sample in Phase 2, selected for their extensive use of MIS techniques (general surgery, gynecology, orthopedics, otorhinolaryngology and head and neck surgery, and urology), and had at least 10 years of experience with MIS. Recruitment continued until we had at least 60 participants, with at least 5 from each of the 5 surgical fields mentioned above. Participating expert surgeons had, on average, 16.7 (SD 9.6) years of experience with MIS. The expert surgeons were not compensated for their participation.

#### Procedure

Each expert was invited to 1 session lasting about 50 minutes. At the session, participants first completed the VR technical aptitude test. They then provided demographic information (age, gender, dominant hand, and surgical specialty), and reported their previous experience using laparoscopic simulators and playing video games as in the previous phases.

### Validation and Analyses

Following the contemporary framework of validity [44,45], we collected evidence on relationships between the 2 novel tests and other variables. In Phase 1, we collected evidence on the relationship between the selection tests and 4 established psychometric instruments measuring similar and different constructs (convergent and discriminant evidence). Toward this end, we used Pearson correlations to assess the associations between the novel selection test scores and scores on the 4 established psychometric instruments (the PPT, MRT, RAPM, and mini-IPIP). Looking first at the VR technical aptitude test, to evaluate convergent evidence for validity, we computed the correlations between interns’ scores on this test and their scores on 2 established psychometric instruments assessing similar competencies—the PPT and the MRT. Those correlations are expected to be relatively strong. We then evaluated discriminant evidence for the VR test by computing the correlations between scores on this test and on 2 established psychometric instruments assessing different competencies—the RAPM and the mini-IPIP. Those correlations are expected to be relatively weak. Turning to the GBA, this time, correlations with the RAPM and the mini-IPIP were used to assess convergent evidence, and correlations with the PPT and the MRT were used to assess discriminant evidence. Finally, correlations between scores on the 2 novel tests were calculated as another source of discriminant evidence.

In Phase 2, we collected evidence for test-criterion relationships based on correlations between the novel selection test scores and measures of performance on each relevant criterion extracted from residency performance evaluations. To do so, we first averaged, for each participant, the scores for each dimension in the evaluation form provided by all supervisors who evaluated that resident (at least 3 supervisors for each participant, as described above). This process resulted in a set of 16 criterion scores for each participant. As another preliminary step, we calculated the average of all dimension scores, excluding the technical skills and general performance dimensions, to produce a mean performance evaluation without technical skills. We then turned to our main analysis—namely, examining the correlations between the 2 selection test scores and the 17 criterion assessments (the 16 dimension scores and the mean performance evaluation without technical skills). We used Pearson correlations to assess the relationships between participants’ scores on the selection tests and their criterion scores. Due to the hierarchical structure of the data (in that residents are nested in different surgical fields and departments), the correlations were calculated separately for each surgical field, and then their weighted mean was calculated across the surgical fields. The correlations were conducted to evaluate prespecified, theory-driven hypotheses regarding the relationships between each selection test and specific performance criteria. Because each analysis addressed a distinct, theory-driven hypothesis and the outcomes were conceptually related rather than independent, formal familywise error correction was not applied [60,61]. Results were interpreted in light of the overall pattern of associations rather than on the basis of isolated statistically significant findings. We expected relatively high correlations between each selection test and its relevant criteria (ie, between technical aptitude test scores and the technical skills criterion, and between GBA scores and all other criterion scores). In addition, we expected that the GBA would be correlated more strongly with those criteria capturing competencies similar to those assessed by the GBA (eg, learning ability and decision-making) than those capturing competencies not assessed by the GBA (eg, communication with patients and their families and communication with medical staff and teamwork).

Based on the data collected in Phase 2, we also examined incremental evidence of validity. To this end, we estimated nested multilevel (random-intercept) models to assess whether each selection test explained unique variance in its relevant residency performance criterion beyond the other test. Residents were modeled as nested within surgical fields. For each criterion, we compared models including a single predictor with models including both predictors using likelihood ratio tests. Changes in model fit and marginal *R*² (ie, the variance explained by the fixed effects only) were examined to evaluate the incremental contribution of each selection test beyond the other test. We expected that each test would demonstrate a unique and statistically significant contribution to its theoretically aligned performance criterion beyond the alternative selection test.

Following Phase 3, we used the VR-based technical aptitude test scores obtained in all 3 phases to examine evidence based on the relationship between test performance and training level (expert-novice differences). For this purpose, we analyzed the VR technical aptitude test scores of expert surgeons obtained in Phase 3 alongside the comparable data obtained from interns and residents in Phases 1 and 2, using a 1-way between-subjects ANOVA. We hypothesized that there would be a positive correlation between training level and technical aptitude test scores, such that groups with more advanced training would have higher scores (ie, experts would score higher than residents and residents higher than interns).

Finally, based on the data of residents in Phase 2, we conducted a fairness analysis to determine whether the selection test discriminates on the basis of gender. Group bias is considered present if the predicted values of the criterion based on test scores differ between the examined groups (ie, if the same selection test score predicts different criterion scores for individuals from the different groups of interest). Toward this end, we conducted a multiple regression analysis for each selection test which included the relevant criterion score (technical skills for the technical aptitude tests and the mean performance evaluation without technical skills for the GBA) as a dependent variable, and test scores and gender as predictors [62,63]. Because evaluators differed between surgical specialties, criterion scores were standardized within each specialty to minimize potential rater effects prior to conducting the regression analyses. Differential prediction was evaluated by testing for slope and intercept differences between the gender groups, and by testing for systematic deviations from the common regression line (evidence for bias increases as the sum of deviations for a specific gender group grows larger). We hypothesized that, although mean gender differences in test scores might be observed, the relationship between test scores and performance criteria would not differ by gender, indicating no evidence of differential prediction.

To examine whether differential prior exposure influenced fairness-related inferences, we also conducted sensitivity analyses in which the fairness regression models were reestimated, including prior laparoscopic simulator experience and prior video game experience as covariates. Based on prior findings indicating modest associations between simulator and/or video game experience and performance on the VR technical aptitude test [46], and between video game experience and benchmark performance [47], the primary specification included simulator experience and video game experience as covariates in the VR technical aptitude model, and video game experience as a covariate in the GBA model.

All tests were 2-sided, and the level of statistical significance was set to .05. Statistical analyses were performed using R (version 4.3.2; R Core Team; R Foundation for Statistical Computing).

## Results

### Participant Characteristics

The demographic characteristics of the 76 interns, 75 residents, and 65 expert surgeons who participated in the study are presented in Table 2.

**Table 2.** Demographic characteristics of the participants.

Characteristic	Interns (n=76)	Residents (n=75)	Experts (n=65)
Age (years), mean (SD)	26.8 (4.0)	34.2 (4.8)	54.5 (9.8)
Sex (female), n (%)	31 (41)	23 (31)	16 (25)
Left dominant hand, n (%)	6 (8)	5 (7)	8 (12)
Surgical specialty, n (%)			
General surgery	—^a^	19 (25)	18 (28)
Gynecology	—	17 (23)	12 (18)
Orthopedics	—	16 (21)	16 (25)
Otorhinolaryngology and head and neck surgery	—	13 (17)	12 (18)
Urology	—	10 (13)	7 (11)
Experience with MIS^b^ simulators, n (%)			
No experience	59 (78)	40 (53)	7 (11)
Little experience	16 (21)	30 (40)	19 (29)
Moderate experience	1 (1)	3 (4)	27 (42)
Considerable experience	0 (0)	2 (3)	11 (17)
Very extensive experience	0 (0)	0 (0)	1 (2)
Experience with video games, n (%)			
No experience	10 (13)	18 (24)	22 (34)
Little experience	19 (25)	30 (40)	31 (48)
Moderate experience	26 (34)	18 (24)	8 (12)
Considerable experience	14 (18)	6 (8)	3 (5)
Very extensive experience	7 (9)	3 (4)	1 (2)

a Not applicable.

b MIS: minimally invasive surgery.

In what follows, we present the results according to their relevance for the different types of validity evidence and for fairness.

### Convergent and Discriminant Evidence for Validity: Phase 1

Means and SDs of scores on the 4 established instruments are shown in Table S2 in the Multimedia Appendix 1. Correlations between scores on the 2 novel selection tests and the 4 established instruments are presented in Table 3.

**Table 3.** Correlations between scores on the 2 novel selection tests and the 4 established psychometric instruments (Phase 1).

Established instrument	VR^a^ technical aptitude test			GBA^b^ of cognitive abilities and personality		
	*r*	95% CI	*P* value	*r*	95% CI	*P* value
PPT^c^	0.33	0.11 to 0.52	.003	0.12	−0.11 to 0.33	.30
MRT^d^	0.59	0.42 to 0.72	<.001	0.38	0.17 to 0.56	.001
RAPM^e^	0.32	0.10 to 0.51	.005	0.54	0.36 to 0.68	<.001
mini-IPIP^f^ scale						
Extraversion	−0.08	−0.30 to 0.15	.50	0.02	−0.21 to 0.25	.86
Agreeableness	−0.13	−0.34 to 0.10	.26	−0.12	−0.33 to 0.11	.30
Conscientiousness	0.20	−0.03 to 0.41	.08	0.29	0.07 to 0.48	.01
Neuroticism	−0.21	−0.42 to 0.02	.07	−0.25	−0.45 to −0.03	.03
Openness	0.01	−0.22 to 0.24	.93	0.14	−0.09 to 0.35	.23

a VR: virtual reality.

b GBA: game-based assessment.

c PPT: Purdue Pegboard Test.

d MRT: Mental Rotation Test.

e RAPM: Raven Advanced Progressive Matrices.

f mini-IPIP: short version of the International Personality Item Pool.

As expected, scores on the PPT were significantly correlated with scores on the technical aptitude test, but not with scores on the GBA. Scores on the MRT were significantly correlated with scores on both the technical aptitude test and the GBA, although the correlation with the former was considerably stronger (0.59 vs 0.38). For the RAPM and the mini-IPIP, we obtained an inverse pattern, again largely in keeping with our expectations. Specifically, scores on the RAPM were significantly correlated with scores on both the technical aptitude test and the GBA, but this time the correlation with the GBA was substantially stronger (0.54 vs 0.32). For the mini-IPIP, the picture was somewhat more complicated. While none of the 5 scales of the mini-IPIP were significantly correlated with scores on the technical aptitude test, the correlations with 2 of these scales, for conscientiousness and neuroticism, were marginally significant (conscientiousness: *r*_74_=0.20, 95% CI −0.03to 0.41, *P*=.08); neuroticism: *r*_74_=−0.21, 95% CI −0.42to 0.02, *P*=.07). However, scores for both of those scales correlated significantly with scores on the GBA. In addition, we found a moderately significant correlation between scores on the technical aptitude test and the GBA (*r*_74_=0.37, 95% CI 0.16-0.55, *P*=.001).

Overall, the correlations between scores on the novel tests and established psychometric instruments measuring similar competencies were stronger than the correlations with established psychometric instruments measuring different competencies. Likewise, correlations between each of the established instruments and the relevant novel selection test measuring similar competencies were higher than those with the selection test measuring different competencies. Therefore, the convergent and discriminant evidence presented supports the validity of the 2 novel selection tests.

### Evidence for Test-Criterion Relationship and Incremental Contribution: Phase 2

To evaluate the evidence for the test-criterion relationship, we first calculated for each resident 16 criterion scores, as described above. Means and SDs of the criterion scores are shown in Table S3 in the Multimedia Appendix 1. The intercorrelations among the 16 performance criteria ranged from 0.21 to 0.89 (mean *r* 0.55), indicating moderate shared variance across domains. As noted above, the number of raters evaluating each resident ranged from 3 to 4, depending on the surgical field. Therefore, to ensure the quality of the criterion scores, we first assessed interrater reliability using the intraclass correlation coefficient (ICC) reliability index. The ICC was calculated separately for each dimension and each surgical field, based on a mean rating (3≤k≤4) consistency 2-way mixed-effects model [64]. The ICC estimates and their 95% CIs are shown in Table S4 in the Multimedia Appendix 1. Overall, the ICC estimates were high, ranging from 0.46 to 0.96 with a median value of 0.78, indicating good interrater reliability of the criterion evaluations.

Then, we examined the correlations between the 2 selection test scores and the 17 criterion assessments (the 16 dimension scores and the mean performance evaluation without technical skills). As noted above, the correlations were first calculated separately for each surgical field (results for each specialty are shown in Table S5 in the Multimedia Appendix 1). We then calculated their weighted mean across the surgical fields. The mean correlations are presented in Table 4.

**Table 4.** Correlations between scores on the 2 selection tests and the criteria scores (Phase 2).

Criterion	VR^a^ technical aptitude test			GBA^b^ of cognitive abilities and personality		
	*r*	95% CI	*P* value	*r*	95% CI	*P* value
Medical knowledge	0.20	−0.03 to 0.41	.10	0.40	0.18 to 0.58	<.001
Technical skills	0.61	0.44 to 0.74	<.001	0.32	0.09 to 0.51	.007
Communication with patients and their families	0.05	−0.18 to 0.28	.68	0.23	0.00 to 0.44	.05
Communication with medical staff and teamwork	0.13	−0.11 to 0.35	.28	0.24	0.01 to 0.45	.04
Integrity	0.13	−0.11 to 0.35	.28	0.36	0.14 to 0.55	.002
Diligence	0.13	−0.11 to 0.35	.28	0.32	0.09 to 0.51	.007
Learning ability	0.15	−0.09 to 0.37	.21	0.41	0.19 to 0.59	<.001
Decision-making and problem-solving	0.28	0.05 to 0.48	.02	0.40	0.18 to 0.58	<.001
Self-criticism and ability to learn from mistakes	0.16	−0.08 to 0.38	.18	0.33	0.10 to 0.52	.005
Thoroughness	0.06	−0.17 to 0.29	.62	0.15	−0.09 to 0.37	.21
Organization and planning	−0.07	−0.30 to 0.17	.56	0.24	0.01 to 0.45	.04
Physical and mental endurance	0.24	0.01 to 0.45	.04	0.30	0.07 to 0.49	.01
Stress tolerance	0.30	0.07 to 0.49	.01	0.32	0.09 to 0.51	.007
Creativity and cognitive flexibility	0.27	0.04 to 0.47	.02	0.34	0.11 to 0.53	.004
Motivation	0.15	−0.09 to 0.37	.21	0.33	0.10 to 0.52	.005
General assessment	0.19	−0.04 to 0.40	.11	0.46	0.25 to 0.63	<.001
Mean performance evaluation without technical skills	0.17	−0.07 to 0.39	.16	0.43	0.21 to 0.60	<.001

a VR: virtual reality.

b GBA: game-based assessment.

As in Phase 1, as a secondary analysis, we calculated the correlation between residents’ scores on the technical aptitude test and the GBA. This correlation was again found to be moderate and significant (*r*_69_=0.40, 95% CI 0.18-0.58, *P*<.001).

As expected, scores on the technical aptitude test were strongly and significantly correlated with the technical skills criterion. In addition, significant (though weak) correlations were found between technical aptitude test scores and 3 criteria: endurance, stress tolerance, and creativity and cognitive flexibility. In contrast, GBA scores were significantly correlated with all criteria (including the technical skills criterion) except thoroughness, with the strongest correlations emerging for medical knowledge, learning ability, decision-making and problem-solving, and general assessment of performance.

Given the moderate intercorrelation between the 2 selection tests and their overlapping associations with several performance criteria, incremental validity analyses were conducted to examine whether each test accounted for unique variance in its theoretically relevant performance criterion above and beyond the other assessment. Specifically, we examined whether the VR-based technical aptitude test uniquely predicted technical skills evaluations, and whether the GBA uniquely predicted overall performance excluding technical skills. Nested multilevel (random-intercept) models were compared using likelihood ratio tests. For technical skills, adding the VR-based technical aptitude test significantly improved model fit (*χ*²_1_=21.18, *P*<.001). In the full model, the VR-based test remained a significant predictor while controlling for GBA scores (*β*=0.03, SE=0.01, 95% CI 0.01-0.05), with marginal *R*² increasing from 0.119 to 0.363. Conversely, for performance excluding technical skills, adding GBA scores significantly improved model fit (*χ*²_1_=8.61, *P*=.003). In the full model, the GBA remained a significant predictor while controlling for VR-based technical aptitude test scores (*β*=0.02, SE=0.01, 95% CI 0.01-0.04), with marginal *R*² increasing from 0.091 to 0.183. These findings indicate that each assessment contributes unique variance to its domain-relevant performance outcome.

Overall, the correlation between scores on the technical aptitude test and its theoretically relevant criterion (ie, technical skills) was substantially stronger than that between GBA scores and the same criterion. Conversely, correlations between GBA scores and performance criteria excluding technical skills were generally stronger than those observed for the technical aptitude test, particularly for the general assessment of performance and the mean performance evaluation without technical skills. At the same time, both tests exhibited significant associations with some nontarget criteria, indicating that the constructs assessed by the 2 tests are partially overlapping rather than fully distinct. The incremental validity analyses further clarified this pattern: the VR-based technical aptitude test uniquely predicted technical skills above and beyond GBA scores, while the GBA uniquely predicted broader nontechnical performance beyond the VR-based test. Together, these findings suggest that the 2 assessments are related but not redundant, and that each contributes distinct and complementary variance to domain-relevant performance outcomes. Accordingly, the pattern of test-criterion relationships contributes to the accumulating evidence regarding the construct validity of both selection tests within Messick’s framework.

### Evidence for Relationship With Training Level: Phases 1, 2, and 3

**Figure 2.**
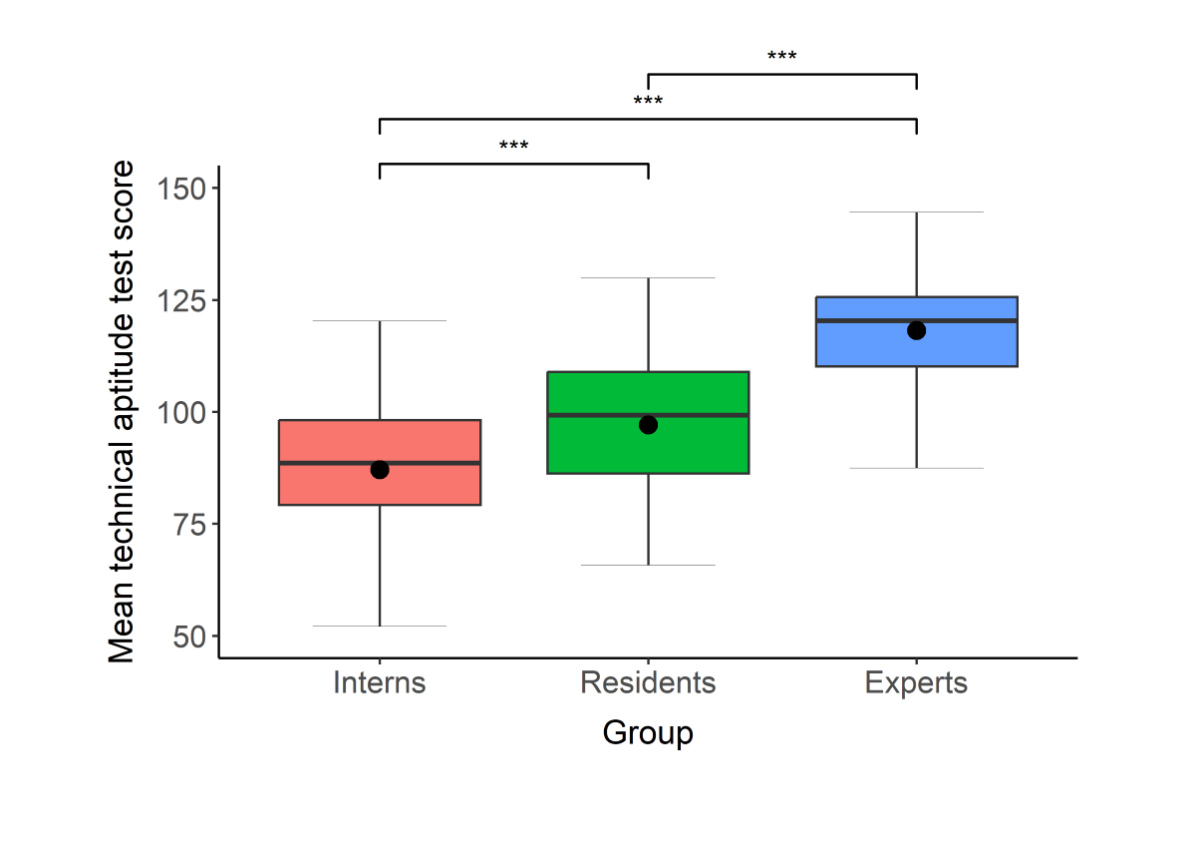
Scores of interns, residents, and expert surgeons on the virtual reality technical aptitude test. Higher scores indicate greater technical aptitude. Within each box, the horizontal bar indicates the median, the circle indicates the mean, and the lower and upper boundaries indicate the first and third quartiles. The vertical lines outside the boxes (whiskers) indicate values within 1.5x the IQR from the upper to lower quartile (or the minimum and the maximum if within 1.5x the IQR of the quartiles). ***, *P*<.001.

We compared the technical aptitude scores of participating interns, residents, and experts via a 1-way between-subjects ANOVA, followed by post hoc comparisons using the Tukey honestly significant difference test. The mean scores of the 3 groups are presented in Figure 2. As expected, we found statistically significant differences between the groups in technical aptitude test scores (*F*_2,211_=72.1, *P*<.001, $\eta^{2}=0.41$). In post hoc comparisons using the Tukey honestly significant difference test, the mean score of the expert surgeons (mean 118.2, SD 13.1) was significantly higher than the mean scores both of residents (mean 97.1, SD 16.1, *P*<.001, difference 95% CI 14.8-27.2) and of interns (mean 87.1, SD 30.9, *P*<.001, difference 95% CI 24.9-37.3). In addition, the residents’ mean score was significantly higher than that of the interns (*P*<.001, difference 95% CI 4.1-16.0). We also separately compared the scores of the 3 groups for each of the performance parameters assessed by the simulator (success rate, time, number of mistakes, path length, and percent of time within scope; refer to Table S6 and Figure S1 in the Multimedia Appendix 1). The pattern of results obtained when examining each parameter separately was similar to the pattern obtained for the final technical aptitude test scores, suggesting that group differences exist for all performance parameters.

Finally, we also calculated correlations between technical aptitude test scores and more specific measures of surgical experience: number of years in surgical training among residents, and number of years of experience with MIS among the expert surgeons. Both correlations were significant (residents: *r*_73_=0.26, 95% CI 0.03-0.47, *P*=.02; experts: *r*_63_=0.50, 95% CI 0.29-0.67, *P*<.001), providing further support for the relationship with training level.

### Gender Bias Analyses: Phase 2

Based on the data obtained from residents in Phase 2, we conducted a fairness analysis to assess the possibility of differential prediction with respect to gender. First, we compared the scores obtained separately by males and females in the selection tests and in the relevant evaluation rating criteria (technical skills evaluation ratings and mean evaluation ratings without technical skills). We found that males scored significantly higher than females in both selection tests (technical aptitude test scores: mean difference 10.7, SD 15.3, 95% CI 2.75‐18.65, *t*_69_=2.67, *P*=.009, Cohen *d*=0.7; GBA scores: mean difference 9.0, SD 18.0, 95% CI 0.16‐17.84, *t*_69_=2.03, *P*=0.046, Cohen *d*=0.5). In the relevant evaluation ratings (the criteria), we found a significant difference between males and females in the technical skills evaluation ratings (mean difference 0.5, SD 0.6, 95% CI 0.11‐0.90, *t*_69_= 2.27, *P*=.03, Cohen *d*=0.8), but only a marginally significant difference in mean evaluation ratings without technical skills (mean difference 0.2, SD 0.4, 95% CI −0.02to 0.50, *t*_69_=1.87, *P*=.07, Cohen *d*=0.5).

Next, we conducted 2 multiple regression analyses. The first of these included technical aptitude test scores (a continuous variable), gender (a dummy variable), and their interaction as predictors, and the technical skills criterion as the dependent variable. The second included GBA scores (a continuous variable), gender (a dummy variable), and their interaction as predictors, and the mean evaluation ratings without the technical skills criterion as the dependent variable (refer to Table S7 in the Multimedia Appendix 1 for the regression statistics). In both regression analyses, the correlation between the selection test and the criterion was significant (in the first regression, *t*_64_=3.6, *P*<.001; in the second regression, *t*_64_=2.70, *P*=.009). However, the effects of gender (in the first regression: *t*_64_=0.25, *P*=.80; in the second regression: *t*_64_=−0.006, *P*=.99) and of the test-gender interaction (in the first regression: *t*_64_=0.76, *P*=.45; in the second regression: *t*_64_=−0.44, *P*=.66) were not significant. Prior simulator and video game experience were modestly associated with relevant selection test scores (*r*=0.11-0.16; Table S8 in the Multimedia Appendix 1). Sensitivity analyses incorporating prior simulator experience and prior video game experience (technical aptitude test model) and prior video game experience (GBA model) as covariates yielded substantively similar conclusions, meaning that the inclusion of differential prior exposure did not materially alter the pattern of regression coefficients or the absence of significant gender-by-test interactions (results are provided in Table S9 in the Multimedia Appendix 1). In other words, the predictive relationship between test scores and performance criteria was comparable for men and women even after accounting for prior simulator and gaming experience. Thus, the regression analyses found no evidence for gender bias.

**Figure 3.**
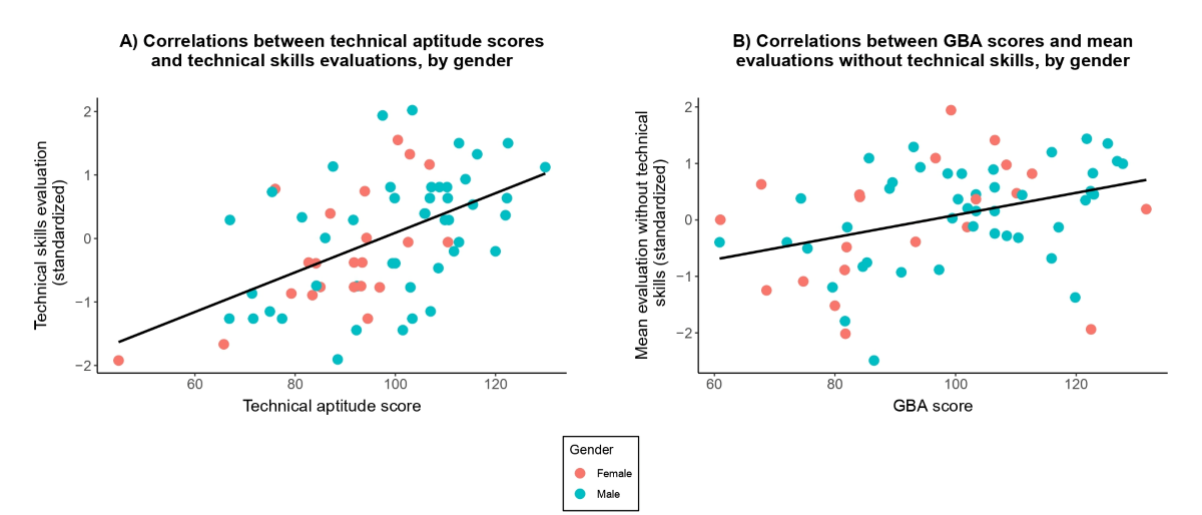
Scatter plots of the correlations between scores on the selection tests and the evaluation criteria by gender. The black lines indicate the common regression lines for both genders. Observations above the regression lines represent positive deviations from the regression lines, and observations below the regression lines represent negative deviations. GBA: game-based assessment.

As a final step, we examined the deviations of female and male residents from the common regression lines (Figure 3). The sum of the deviations for both genders in both regression models was close to zero (since for both genders, negative and positive deviations largely canceled each other out). Thus, this analysis also suggests that the prediction is not biased toward one of the genders. Overall, these findings do not support the existence of bias in either of the 2 selection tests.

## Discussion

### Principal Findings

This study examined evidence based on relationships with other variables for validation of 2 simulation-based digital assessments—a VR-based technical aptitude test and a GBA of cognitive abilities and personality characteristics—proposed for use in surgical residency selection.

Consistent with our hypotheses, the VR-based technical aptitude test demonstrated construct-consistent patterns of association. Scores were significantly correlated with established psychometric measures of dexterity and visuospatial ability, and with concurrent supervisor evaluations of technical skills among residents. In addition, scores increased systematically with level of surgical experience, differentiating between interns, residents, and expert surgeons. These findings support the interpretation that the VR test captures psychomotor and perceptual abilities relevant to surgical performance, and they reinforce the underlying assumption that higher scores reflect greater technical aptitude and, consequently, stronger technical performance during surgical training.

Similarly, the GBA demonstrated theoretically aligned associations. GBA scores were significantly related to established measures of intelligence and personality, and to supervisor-rated nontechnical performance dimensions in residency. As expected, the findings supported convergent and discriminant patterns consistent with the underlying construct: stronger associations were observed with competencies conceptually aligned with the GBA (eg, in Phase 1: neuroticism, which is related to stress tolerance, and conscientiousness, which is related to thoroughness; in Phase 2: decision-making and problem-solving, learning ability, creativity and cognitive flexibility, and stress tolerance) than with less related interpersonal dimensions (eg, in Phase 1: extraversion and agreeableness, which are related to behavior toward other people; in Phase 2: communication with patients and their families, and communication with medical staff and teamwork). These findings support the interpretation that the GBA measures nontechnical competencies relevant for resident selection, and reinforce the assumption that higher scores reflect higher levels of the assessed competencies.

With respect to fairness, although mean gender differences were observed on both selection tests, there was no evidence of differential prediction. The relationship between test scores and relevant performance criteria did not differ by gender, and findings remained robust after accounting for prior simulator and video game experience. These results suggest that individuals with comparable levels of relevant competencies—regardless of gender—are expected to achieve similar predicted performance outcomes based on test scores, and therefore, there is no evidence that the tests are systematically biased on the basis of gender.

We must note aspects of the findings of this study which suggest that the 2 novel tests do not fully differentiate between the assessment of technical aptitude, on the one hand, and of cognitive abilities and personality on the other. In particular, scores on each test are also correlated with measures not directly related to the construct the test is intended to measure (“construct-irrelevant variance”), suggesting some similarities between the constructs being assessed in the 2 tests. Specifically, scores on the technical aptitude test were significantly correlated with a measure of intelligence (RAPM scores in Phase 1), and with ratings of residents’ decision-making and problem-solving, physical and mental endurance, stress tolerance, and creativity and cognitive flexibility in Phase 2. In addition, scores on the technical aptitude test were correlated with the personality dimensions of conscientiousness and neuroticism at a marginally significant level. With respect to the GBA, scores on this test were significantly correlated with a measure of visuospatial ability (MRT scores in Phase 1) and with technical skills in Phase 2.

Despite this construct-irrelevant variance, however, the stronger associations observed between each test and its theoretically relevant measures, together with the incremental validity findings, indicate that each assessment primarily reflects its intended construct despite partial overlap. Specifically, the incremental analyses demonstrated that the VR-based technical aptitude test uniquely predicted technical skills above and beyond GBA scores, while the GBA uniquely predicted broader nontechnical performance beyond the VR-based test. As such, the findings suggest that the 2 assessments provide complementary, nonredundant information for selection decisions. In this regard, it is also important to note that simulation-based assessments involve complex behavioral tasks that require the simultaneous engagement of multiple competencies [65]. Consequently, some degree of cross-domain association is theoretically expected, as performance in such environments may also draw upon decision-making, stress tolerance, and the ability to learn from feedback. These findings are in line with many previous findings assessing the relationship between performance on surgical simulators and cognitive abilities (eg, intelligence, memory, perceptual speed, and reasoning) [66-68] and personality characteristics (eg, stress tolerance, motivation, conscientiousness, and neuroticism) [69-72], and with studies showing correlations between nontechnical skills (such as decision-making and judgment) and surgical performance [72-74].

### Comparison to the Literature

This study extends 2 previous studies [46,47], which described the development of the 2 novel tests examined here and presented initial evidence for their validity. Although the findings of those studies supported the potential of the tests in the selection of candidates for surgical training, the validity evidence they presented was partial, dealing primarily with content, internal structure, and response process evidence. In addition, although those studies identified gender differences in test scores, they were unable to evaluate potential gender bias because they did not include external criterion measures necessary to assess differential prediction. This study continues the validation process, focusing on evidence derived from relationships between scores on the novel tests and other variables: specifically, relationships with other tests measuring similar and different constructs, test-criterion relationships, and relationships with training level. This study also addresses the issue of gender bias in the tests.

More broadly, our findings contribute to the existing literature examining the use of surgical simulators and game-based assessments for selection in general, as well as specifically for the selection of candidates for surgical training. Although digital simulation-based assessments are considered promising alternatives to traditional selection methods due to their unique advantages—such as the ability to simulate realistic “job-sample” tasks and to generate assessments based on large volumes of objective performance data [18,19,24-26,75]—empirical evidence supporting their use in surgical residency selection remains nascent.

In the broader literature on the use of VR surgical simulators, most validation studies have focused on the use of simulated tasks for training purposes or for assessing the proficiency of residents and practicing surgeons (eg, for feedback, credentialing, or examination) rather than for selection [76-78]. Prior research has demonstrated that performance on VR surgical simulators correlates with operating room performance [19,79,80], that substantial variability exists in learning curves among trainees and surgeons [81-83], and that simulator tasks can effectively discriminate between individuals at different levels of expertise [84-86]. However, as emphasized in the contemporary unified framework of validity described by Messick [44,45], evidence supporting the use of simulated tasks for training or proficiency assessment cannot be automatically generalized to their use for high-stakes selection decisions. It is therefore essential to examine validity evidence specifically in relation to the intended use of simulators as part of a selection process before incorporating them into such decisions.

Evidence specifically supporting the use of simulated tasks for resident selection remains limited [38-42]. Cope and Fenton-Lee [38] found no performance differences between interns who expressed interest in surgical careers and those who did not, suggesting a lack of self-selection. Jardin et al [39] and Salgado et al [42] reported no significant correlations between simulator task performance and traditional academic metrics such as USMLE scores, grades, or interview ratings, leading to the suggestion that technical aptitude assessment may capture competencies not reflected in conventional selection tools. Gallagher et al [40,41] incorporated simulator-based technical tasks into a broader multimethod selection system and reported some validity evidence; however, the evidence pertained to the overall selection system rather than to the simulator-based technical assessment specifically. None of the aforementioned studies used a systematic process to develop a comprehensive test for assessing candidates’ technical aptitude based on accepted psychometric procedures (eg, developing a test blueprint, systematic selection of tasks, and developing a scoring system), or provided significant validity evidence for the use of surgical simulators in the selection process (test content, response process, internal structure, relationships to other variables, and consequences) [43]. In addition, most of these studies used MIS simulators to assess candidates for higher surgical education or surgical fellowships, so their assessment of technical skills is not applicable to candidates without previous surgical experience. In this context, our previous [46] and current studies address this gap by systematically developing and validating a simulation-based technical aptitude assessment for candidates without prior surgical experience.

As GBAs are still relatively new, only a limited number of studies have examined their potential for assessing cognitive abilities and personality characteristics in hiring and recruitment contexts [87-92]. Simons et al [87] developed a GBA to assess aspects of general intelligence and showed associations with established intelligence measures. Wiernik et al [88] designed and validated a GBA to assess cognitive and noncognitive competencies relevant to cyber occupations in the US Air Force, presenting evidence related to content (subject-matter expert input), internal structure (reliability and factor analysis), and relationships with other variables (convergent and discriminant evidence). Similarly, Landers et al [89] developed a theory-driven GBA of general cognitive ability for personnel selection, and revealed evidence for internal consistency, test-criterion relationships with job performance outcomes, and fairness across demographic groups. Haizel et al [90], Quwaider et al [91], and van Lankveld et al [92] explored the use of video game behaviors to infer five-factor personality traits, and provided only preliminary evidence regarding associations with self-report personality inventories. Collectively, this body of work provides initial support for the feasibility of designing game-based environments to assess cognitive abilities, job-relevant competencies, and personality traits for recruitment and occupational screening purposes. However, those studies were conducted in corporate, military, or experimental settings and did not apply game-based assessment to medical education or surgical residency selection. Accordingly, the combined findings of our earlier [47] and present validation studies represent the first systematic examination of the use of a game-based assessment to evaluate cognitive abilities and personality characteristics among candidates for surgical training, and to provide validity evidence supporting its use in surgical residency selection.

In addition, the findings of this study can be situated within the broader literature on the assessment of cognitive abilities and personality traits in the context of selection for surgical training. Traditionally, resident selection processes have relied on proxies such as curricula vitae, letters of recommendation, and unstructured interviews, as well as standardized cognitive measures (eg, academic examinations) [8,93]. Other approaches have included self-report questionnaires assessing personality traits, emotional intelligence, and grit. However, there is currently no consistent evidence that these methods substantially improve the selection of surgical residents [4,17]. In contrast, the GBA examined in this study is specifically designed to operationalize relevant cognitive and nontechnical competencies through interactive tasks that capture dynamic performance indicators (eg, response patterns and learning curves), capturing competencies that have been identified as important for surgical training (eg, decision-making, stress tolerance, and cognitive flexibility) [1-7]. As such, the GBA examined here reflects contemporary assessment approaches that prioritize behavior-based evidence and more realistic task contexts [29].

### Gender Differences

Although we found no evidence of psychometric bias with respect to gender, significant mean differences in selection test scores were observed, with males scoring higher on both tests. It is possible that the gender differences in test scores found in our study and in our 2 previous validation studies [46,47] stem from the specific set of competencies assessed by the tests, or from the format in which the tests were administered. Various studies on sex and gender differences suggest that males and females tend to perform better on different kinds of challenges or tasks. For example, on average, men have been found to score significantly higher than women in tasks involving visuospatial perception and technical skills [75,81,84,94-102], and there is some evidence for a male advantage in intelligence tests [95]. For their part, women are thought to excel (again, on average) in tasks that require verbal abilities [95] and interpersonal skills [103,104]. The tests examined in this study were procedural and did not require verbal ability. In addition, the GBA examined in this study does not assess interpersonal skills, teamwork, leadership, integrity, and other cognitive abilities and personality characteristics relevant for selecting surgical residents, some of which might favor women. Therefore, it is possible that a more comprehensive selection process, including a broader assessment of nontechnical and verbal skills, would decrease or eliminate the gender difference found here. Future studies should examine whether other types of GBAs, such as gamified situational judgment tests [25], or other assessment methods, could lead to improvements in gender parity.

Probing deeper into the gender differences found in this study, these may be influenced, at least in part, by situational, environmental, or experiential factors. Prior exposure to video gaming, technical hobbies, and early engagement with digital tools has been proposed as an important contributor to gender differences in performance on technology-mediated and spatially demanding tasks [105-108]. Such exposure may influence familiarity with the testing format, but it may also contribute to the development of relevant cognitive and procedural skills over time [109,110]. Thus, observed differences could reflect experiential pathways (including broader patterns of socialization and access to opportunities) rather than inherent ability differences, and should therefore be considered when interpreting mean score disparities in VR-based or game-based assessments. Consistent with this perspective, findings from our 2 prior studies indicated that a portion of the gender difference in technical aptitude scores was explained by prior self-reported simulator and video game experience [46], and that the gender difference in GBA performance was largely accounted for by differences in self-reported video game experience [47]. In this study, adjusting for these exposure variables did not affect our findings regarding differential prediction or psychometric fairness. Nonetheless, the mean score differences we found may partly reflect differences in prior experience and opportunity structures. Future research should examine these mechanisms more directly, for example, by incorporating objective measures of prior exposure, experimentally manipulating practice opportunities, or evaluating whether structured familiarization procedures before testing attenuate group-level disparities.

Another possibility, not mutually exclusive with the first, is that the gender differences we found reflect, at least to some extent, a “stereotype threat” effect. According to this explanation, negative stereotypes associated with sex- or gender-typical performance could lead to performance-related anxiety in members of that gender, which ultimately diminishes their performance [95]. Given that surgery is still a male-dominated field, it is possible that this phenomenon affected, to some degree, the performance of female participants in our study. In addition, to the extent that stereotype threat operated among the residents in our sample, this could have affected their performance both in the selection tests and more broadly during their residency, creating a false correlation between their test scores and their performance evaluations. This hypothesis is supported by evidence that stereotype threat indeed may have an effect on surgical trainees [111,112].

Finally, although we found no evidence for gender bias in the tests in the psychometric sense, we recognize that the absence of statistical bias does not resolve all fairness concerns in high-stakes selection contexts. Our analyses indicate that test scores predict relevant performance criteria similarly for men and women; however, differences in average scores may still result in different selection rates when score-based cutoffs are applied. It is therefore important to distinguish between statistical fairness, which concerns the measurement properties of a test, and the practical consequences of its use in selection, which may affect representation even when psychometric bias is absent.

From a measurement perspective, selection tools that meet accepted standards of validity and fairness can improve the accuracy of selection decisions. At the same time, questions related to equity, diversity, and workforce composition involve institutional and policy considerations that extend beyond what psychometric validation alone can address and relate to how selection tools are implemented in practice.

In this broader context, expanding selection processes to include a more comprehensive assessment of nontechnical competencies, such as interpersonal skills, may enhance the overall validity of selection decisions and help mitigate differential outcomes associated with specific ability profiles [103,104]. In addition, institutions may choose to pursue representation goals through policy-level decisions that are external to the assessment tools themselves, such as the use of different selection cutoffs, separate norms, or targeted allocation strategies. Taken together, these considerations underscore the need to interpret psychometric fairness findings cautiously and within a wider applied and societal context.

### Implications

Currently, most surgical programs select residents using traditional methods, whose relationships with later clinical and operative performance have limited and inconsistent empirical support [10-14]. In this study, we present evidence supporting the potential of a VR technical aptitude test and a GBA for objectively assessing cognitive abilities and personality characteristics for the selection of candidates for surgical training.

The combined use of a VR-based technical aptitude test and a gamified behavioral assessment may offer a structured and standardized approach to evaluating multiple competency domains considered relevant for surgical training [1-7]. The technological features of VR and GBAs allow automated administration and scoring, standardized testing conditions, greater engagement of examinees, and high-resolution behavioral data capture. These characteristics may reduce certain sources of human subjectivity in scoring and enhance feasibility in large-scale contexts. In addition, the integration of assessment results across technical, cognitive, and personality-related domains may provide selection committees with a broader representation of candidate performance, offering an in-depth understanding of each candidate’s strengths and weaknesses. Such information could potentially support more informed and structured decision-making, for example, by helping selection committees consider the fit between a candidate’s profile and the characteristics of a given training program. Assessment results may also help educators identify areas where incoming residents might benefit from targeted supervision or skills development. At the program level, aggregated data could contribute to ongoing evaluation of training priorities and curricular design.

The importance of developing and validating structured assessment tools is particularly salient in the current context of surgical education, which faces challenges including work-hour restrictions, increasing technological complexity, and growing economic pressures to enhance efficiency in the operating room [8]. Within this landscape, strengthening the evidentiary foundation of selection processes represents a critical priority. The findings of this study contribute to the cumulative process of validity evidence generation for technology-enhanced assessments in surgical education. The conceptual framework and validation approach may also inform the development of technology-enhanced assessments in other medical specialties and professional domains.

As a final note, we acknowledge that the extent to which the advantages of the proposed tools translate into improved decision-making remains to be determined empirically, particularly in light of the current absence of longitudinal evidence linking pretraining assessment scores to subsequent performance during residency. Future research should extend this work through longitudinal designs, cross-program collaborations, and the development of more standardized performance criteria across training institutions.

### Research Limitations and Future Directions

This study examines a comprehensive assessment of technical aptitude, cognitive abilities, and personality characteristics. Other key strengths include the breadth of the validity evidence provided and the large sample of interns, residents, and expert surgeons.

This study also has limitations. First, while our sample was large and included doctors with varying levels of experience, all participants came from one country, which limits the generalizability of the results. However, the competencies we assessed, such as technical skills in laparoscopic procedures, are fundamental aspects of surgical training and are typically standardized across medical education programs worldwide. This standardization, along with common international guidelines and best practices in surgical training, suggests that these competencies are unlikely to be distributed differently among doctors from different countries. Therefore, we believe our findings still provide valuable insights relevant to a broader international context.

Second, residency performance outcomes were based on supervisor ratings, which are inherently subjective and may be influenced by rater-related biases, including halo effects and lack of full independence among raters working within the same clinical environment. Although each resident was evaluated by multiple supervisors (3 or 4), and interrater reliability was high, complete independence of ratings cannot be assumed within a shared clinical environment where supervisors interact regularly. Indeed, the intercorrelations among the 16 performance criteria ranged from 0.21 to 0.89 (mean *r* 0.55), indicating moderate shared variance across domains. This pattern suggests that the evaluations may partly reflect an overall impression of resident performance. That said, the variability in both correlation magnitudes and mean ratings across criteria indicates that supervisors did not rate all domains uniformly high or low, which would be expected under a strong halo effect. Future research should incorporate additional objective, behavioral, or independently assessed performance indicators to further strengthen criterion-related evidence and reduce the risk of shared method variance.

Third, although this study provided evidence for test-criterion relationships, these data were collected using a concurrent design. Accordingly, based on the current evidence, we cannot conclude definitively that administering these tests to candidates prior to residency would predict their subsequent performance during training. Prospective longitudinal research, in which selection assessments are administered before residency and performance is evaluated at later stages, is required to establish predictive utility in high-stakes selection contexts.

A fourth limitation concerns the proprietary nature of the GBA scoring system. Although the general scoring framework and psychometric properties are described, detailed operational definitions of specific behavioral indicators and exact weighting parameters cannot be publicly disclosed due to contractual and trade secret protections. Consequently, full computational replication of the scoring procedure by independent researchers is not possible. Nevertheless, consistent with established standards for educational and psychological testing [45], this study evaluates score interpretation through relationships with other variables, which remain fully open to empirical scrutiny.

Fifth, the digital format of both selection tests raises the possibility that prior familiarity with gaming or computerized environments may influence performance. Although this study, as well as our 2 previous validation studies [46,47], found only weak associations between self-reported gaming experience and test scores, this potential source of construct-irrelevant variance cannot be entirely excluded. Indeed, some prior research has reported performance advantages for individuals with video game experience in both GBAs [107,108] and surgical simulator tasks [113,114]. Future research should more systematically examine whether prior exposure to digital gaming environments confers an unintended advantage and, if so, explore design-based strategies to mitigate such effects—for example, through standardized familiarization periods, structured practice sessions prior to scored tasks, interface simplification, or task designs that reduce construct-irrelevant variance arising from digital interface familiarity.

Finally, the gamified test presented in our study does not assess all cognitive abilities and personality characteristics relevant to the selection of surgical residents. In particular, it does not include important competencies such as interpersonal skills, teamwork, leadership, and integrity. Future studies should examine whether additional assessment methods could complement the proposed tools in capturing these competencies. Established approaches in selection research include traditional situational judgment tests, structured self-report questionnaires, and simulation-based assessments with trained observers rating interpersonal behaviors [115]. In parallel, recent technological developments offer promising extensions, such as gamified situational judgment tests [25], as well as emerging approaches using machine learning to model interpersonal behaviors from rich behavioral data [116]. Integrating such methods may enable a more comprehensive and ecologically valid assessment of nontechnical competencies relevant to surgical training selection.

### Conclusions

This study presents validity evidence regarding the use of a VR-based technical aptitude test and a game-based assessment of cognitive abilities and personality characteristics in the selection of candidates for surgical training. The findings extend 2 previous validation studies by contributing additional evidence within the domain of relations to other variables, as well as evidence regarding gender fairness. Although this study does not empirically establish downstream effects on residency outcomes, attrition, or patient care, the rigorous validation of structured assessment approaches may represent an important step toward more evidence-informed selection practices. To achieve a more comprehensive evaluation of competencies relevant to surgical training, these assessments may be considered as components of a broader, multimodal selection framework that also includes measures of interpersonal and communication skills.

## Supplementary material

10.2196/82515 Multimedia Appendix 1 Supporting materials for study analyses.

## Abbreviations

GBA: game-based assessment
ICC: intraclass correlation coefficient
mini-IPIP: short version of the International Personality Item Pool
MIS: minimally invasive surgery
MRT: Mental Rotation Test
PPT: Purdue Pegboard Test
RAPM: Raven’s Advanced Progressive Matrices
USMLE: United States Medical Licensing Examination
VR: virtual reality
