# Supplementary material for: Virtual Reality and Gamification for Assessing Technical Aptitude, Cognitive Abilities, and Personality Characteristics in Surgical Residency Selection: Validation Study
Source: JMIR Med Educ. 2026 May 25;12:e82515. doi: 10.2196/82515 (PMC13200778; doi:10.2196/82515)
Supplement: Multimedia Appendix 1 — Supporting materials for study analyses. [file mededu-v12-e82515-s001.docx]

**Appendix**

**Table S1**. The Four Traditional Tests Used in Phase 1 for Evaluation of Convergent and Discriminant Evidence

**Table S2**. Interns’ Scores on the Four Traditional Tests (Phase 1)

**Table S3**. Residents’ Scores on the Residency Evaluation Dimensions (Phase 2)

**Table S4**. ICC Estimates of Evaluation Dimensions by Surgical Field (Inter-Rater Reliability)

**Table S5**. Correlations Between Scores on the Two Selection Tests and the Criteria, by Surgical Field (Phase 2)

**Table S6**. Parameter Scores of Interns, Residents, and Expert Surgeons on the VR Technical Aptitude Test (ANOVA and Post Hoc Comparisons)

**Figure S1**. Distributions of Performance Parameters in the VR Technical Aptitude Test by Group

**Table S7**. Summary of Multiple Regression Analyses for the Evaluation of Gender Bias

**Table S8**. Correlations Between Prior Simulator and Video Game Experience and Selection Test Scores (Phase 2)

**Table S9**. Summary of Multiple Regression Analyses for the Evaluation of Gender Bias (Sensitivity Analyses)

**Table S1.** The Four Traditional Tests Used in Phase 1 for Evaluation of Convergent and Discriminant Evidence.

| Test | Competencies | Test description | Scoring | Psychometric properties |
| --- | --- | --- | --- | --- |
| Purdue Pegboard Test (PPT) | Manual dexterity and bimanual coordination | The pegboard consists of a board with two parallel rows of 25 holes into which cylindrical metal pegs (pins) are placed by the examinee. The test involves a total of four trials (usually, each trial is repeated three times). In the first three trials, the examinee places as many pins as possible in the holes, first with the preferred hand, then with the nonpreferred hand, and finally with both hands, within a 30 second period. In the fourth trial, the examinee uses both hands alternately to construct “assemblies” comprising a pin, a washer, a collar, and another washer. The examinee is instructed to complete as many assemblies as possible within 60 seconds. | In the original test, scores are calculated separately for each trial, based on the average number of parts placed in the board across the three repetitions of each trial. In this study, we also calculated total test scores by scaling and averaging scores for the four trials. | High test–retest reliability (0.81–0.89)^1^ |
| Mental Rotation Test (MRT) | Depth perception and visuospatial ability | The version of the test used in this study (the MRT-A) contains 24 items. In each item, the examinee is presented with a target stimulus figure (a three-dimensional object) on the left, and four other stimulus figures on the right, two of which are rotated versions of the target stimulus. Examinees are asked to identify the two stimuli that match the target stimulus. The 24 items are arranged in two sets of 12 items, and examinees have three minutes for each set. | One point is awarded for each item if both stimulus figures that match the target figure are identified correctly. Therefore, the maximum score obtainable in the test is 24. | High internal consistency (0.88)^2^  High test–retest reliability (0.83)^2^ |
| Raven’s Advanced Progressive Matrices (RAMP) | General intelligence and abstract reasoning | The RAPM is intended for adults with above-average intelligence. The test contains 48 items, presented in two sets (set I – a practice set comprising 12 items, and set II – a test set comprising 36 items). Each item consists of a visual geometric design with a missing piece; examinees are asked to identify which of eight possible options represents the missing piece. Examinees are given 5 minutes for set I and 40 minutes for set II. | One point is given for each correct answer in set II, resulting in a maximum score of 36. | High internal consistency (0.6–0.98, with a median of 0.9)^3,4^  Moderate to high test–retest reliability (0.67– 0.83)^3,4^ |
| Mini International Personality Item Pool (Mini-IPIP) | Five-factor model traits: extraversion, agreeableness, conscientiousness, neuroticism and openness | The mini-IPIP is a 20-item self-report inventory, with four items per big-five trait. In each item subjects are presented with a phrase relating to the subject’s own personality and asked to rate their agreement with this phrase on a 5-point scale (1 = strongly disagree, 5 = strongly agree). | A total score is calculated for each trait by summing the ratings for the four relevant items. Therefore, each trait score ranges from 4 to 20, with higher values indicating higher levels of the trait. | High internal consistency (0.79–0.91)^5^  Moderate to high test–retest reliability (0.68– 0.88)^5^ |

**References:**

1. Buddenberg LA, Davis C. Test–Retest Reliability of the Purdue Pegboard Test. Am J Occup Ther. 2000;54:555–8. https://doi.org/10.5014/AJOT.54.5.555.

2. Vandenberg’ And SG, Kuse AR, Ashton GC, Johnson RC, Mi MP, Rashad MN, et al. Mental rotations, a group test of three-dimensional spatial visualization. Percept Mot Skills. 1978;47:599–604. https://doi.org/10.2466/PMS.1978.47.2.599.

3. Raven JC. Court J. The Advanced Progressive Matrices. In: Manual for Raven’s progressive matrices and vocabulary scales. San Antonio, TX: Harcourt Assessment; 1998.

4. Arthur W, Tubre TC, Paul DS, Sanchez-Ku ML. College-Sample Psychometric and Normative Data on a Short Form of the Raven Advanced Progressive Matrices Test. http://dx.doi.org/101177/073428299901700405. 1999;17:354–61. https://doi.org/10.1177/073428299901700405.

5. Donnellan MB, Oswald FL, Baird BM, Lucas RE. The mini-IPIP scales: tiny-yet-effective measures of the Big Five factors of personality. Psychol Assess. 2006;18:192–203. https://doi.org/10.1037/1040-3590.18.2.192.

**Table S2.** Interns’ Scores on the Four Traditional Tests (Phase 1).

| **Test** | **Mean (SD);  *N* = 76** | **95% CI** | **Range** |
| --- | --- | --- | --- |
| **PPT** |  |  |  |
| Right | 15.3 (1.7) | [14.9, 15.7] | 11.3 – 20.3 |
| Left | 14.5 (1.6) | [14.1, 14.9] | 9.3 – 17.3 |
| Both hands | 12.6 (1.4) | [12.3, 12.9] | 8.0 – 15.3 |
| Assembly | 43.4 (5.4) | [42.2, 44.6] | 33.3 – 57.7 |
| **MRT** | 13.8 (5.0) | [12.7, 14.9] | 3 – 24 |
| **RAPM** | 28.2 (3.7) | [27.4, 29.1] | 19 – 36 |
| **Mini-IPIP scale**: |  |  |  |
| Extraversion | 12.4 (3.5) | [11.6, 13.2] | 6 – 20 |
| Agreeableness | 17.4 (2.5) | [16.8, 18.0] | 11 – 20 |
| Conscientiousness | 15.1 (3.5) | [14.3, 15.9] | 6 – 20 |
| Neuroticism | 10.4 (3.8) | [9.5, 11.3] | 4 – 19 |
| Openness | 15.3 (3.1) | [14.6, 16.0] | 10 – 20 |

**Table S3.** Residents’ Scores on the Residency Evaluation Dimensions (Phase 2).

| **Dimension** | **Mean (SD);  *n* = 75** | **95% CI** | **Range** |
| --- | --- | --- | --- |
| **1. Medical knowledge** | 3.6 (0.8) | [3.4, 3.8] | 1.5 – 5 |
| **2. Technical skills** | 3.6 (0.8) | [3.4, 3.8] | 2 – 5 |
| **3. Communication with patients and their families** | 4.1 (0.8) | [3.9, 4.3] | 1 – 5 |
| **4. Communication with medical staff and teamwork** | 4.1 (0.9) | [3.9, 4.3] | 1 – 5 |
| **5. Integrity** | 4.3 (0.6) | [4.2, 4.4] | 2.5 – 5 |
| **6. Diligence** | 4.0 (0.7) | [3.8, 4.2] | 2 – 5 |
| **7. Learning ability** | 4.1 (0.7) | [3.9, 4.3] | 2.3 – 5 |
| **8. Decision-making and problem-solving** | 3.8 (0.8) | [3.6, 4.0] | 2 – 5 |
| **9. Self-criticism and ability to learn from mistakes** | 3.8 (0.8) | [3.6, 4.0] | 1 – 5 |
| **10. Thoroughness** | 3.8 (0.7) | [3.6, 4.0] | 1.5 – 5 |
| **11. Organization and planning** | 3.8 (0.7) | [3.6, 4.0] | 2 – 5 |
| **12. Physical and mental endurance** | 4.1 (0.7) | [3.9, 4.3] | 2 – 5 |
| **13. Stress tolerance** | 4.0 (0.7) | [3.8, 4.2] | 2 – 5 |
| **14. Creativity and cognitive flexibility** | 3.8 (0.8) | [3.6, 4.0] | 1 – 5 |
| **15. Motivation** | 4.2 (0.7) | [4.0, 4.4] | 2.3 – 5 |
| **16. General assessment** | 3.8 (0.7) | [3.6, 4.0] | 2 – 5 |

**Table S4.** ICC Estimates of Evaluation Dimensions by Surgical Field (Inter-Rater Reliability).

| **Dimension** | **ICC** | ***F*** | **df_1_** | **df_2_** | ***p*** | **95% CI** | |
| --- | --- | --- | --- | --- | --- | --- | --- |
|  |  |  |  |  |  | ***lower*** | ***upper*** |
| **1. Medical knowledge** |  |  |  |  |  |  |  |
| General surgery | 0.84 | 6.08 | 14 | 28 | 0.00003 | 0.61 | 0.94 |
| Gynecology | 0.73 | 3.65 | 16 | 48 | 0.00025 | 0.43 | 0.89 |
| Orthopedics | 0.70 | 3.39 | 15 | 15 | 0.01197 | 0.16 | 0.90 |
| Otorhinolaryngology | 0.78 | 4.48 | 11 | 22 | 0.00136 | 0.41 | 0.93 |
| Urology | 0.82 | 5.42 | 9 | 18 | 0.00116 | 0.46 | 0.95 |
| **2. Technical skills** |  |  |  |  |  |  |  |
| General surgery | 0.90 | 9.80 | 14 | 28 | 0.00000 | 0.76 | 0.96 |
| Gynecology | 0.85 | 6.81 | 16 | 48 | 0.00000 | 0.69 | 0.94 |
| Orthopedics | 0.82 | 5.63 | 15 | 15 | 0.00091 | 0.49 | 0.94 |
| Otorhinolaryngology | 0.76 | 4.17 | 11 | 22 | 0.00214 | 0.36 | 0.92 |
| Urology | 0.78 | 4.46 | 9 | 18 | 0.00342 | 0.34 | 0.94 |
| **3. Communication with patients and their families** |  |  |  |  |  |  |  |
| General surgery | 0.76 | 4.11 | 14 | 28 | 0.00071 | 0.42 | 0.91 |
| Gynecology | 0.71 | 3.48 | 16 | 48 | 0.00041 | 0.40 | 0.88 |
| Orthopedics | 0.92 | 13.16 | 15 | 15 | 0.00001 | 0.78 | 0.97 |
| Otorhinolaryngology | 0.73 | 3.69 | 11 | 22 | 0.00444 | 0.28 | 0.92 |
| Urology | 0.92 | 13.10 | 9 | 18 | 0.00000 | 0.78 | 0.98 |
| **4. Communication with medical staff and teamwork** |  |  |  |  |  |  |  |
| General surgery | 0.90 | 9.62 | 14 | 28 | 0.00000 | 0.75 | 0.96 |
| Gynecology | 0.80 | 5.06 | 16 | 48 | 0.00001 | 0.59 | 0.92 |
| Orthopedics | 0.89 | 8.75 | 15 | 15 | 0.00007 | 0.67 | 0.96 |
| Otorhinolaryngology | 0.81 | 5.17 | 11 | 22 | 0.00052 | 0.49 | 0.94 |
| Urology | 0.79 | 4.85 | 9 | 18 | 0.00216 | 0.40 | 0.94 |
| **5. Integrity** |  |  |  |  |  |  |  |
| General surgery | 0.69 | 3.24 | 14 | 28 | 0.00390 | 0.27 | 0.89 |
| Gynecology | 0.85 | 6.53 | 16 | 48 | 0.00000 | 0.68 | 0.94 |
| Orthopedics | 0.85 | 6.81 | 15 | 15 | 0.00031 | 0.58 | 0.95 |
| Otorhinolaryngology | 0.55 | 2.20 | 11 | 22 | 0.05561 | -0.20 | 0.86 |
| Urology | 0.89 | 8.81 | 9 | 18 | 0.00005 | 0.67 | 0.97 |
| **6. Diligence** |  |  |  |  |  |  |  |
| General surgery | 0.76 | 4.20 | 14 | 28 | 0.00061 | 0.43 | 0.91 |
| Gynecology | 0.46 | 1.87 | 16 | 48 | 0.04885 | -0.12 | 0.78 |
| Orthopedics | 0.79 | 4.79 | 15 | 15 | 0.00217 | 0.40 | 0.93 |
| Otorhinolaryngology | 0.78 | 4.48 | 11 | 22 | 0.00136 | 0.41 | 0.93 |
| Urology | 0.92 | 12.83 | 9 | 18 | 0.00000 | 0.77 | 0.98 |
| **7. Learning ability** |  |  |  |  |  |  |  |
| General surgery | 0.68 | 3.17 | 14 | 28 | 0.00450 | 0.25 | 0.89 |
| Gynecology | 0.64 | 2.80 | 16 | 48 | 0.00303 | 0.25 | 0.86 |
| Orthopedics | 0.83 | 5.72 | 15 | 15 | 0.00084 | 0.50 | 0.94 |
| Otorhinolaryngology | 0.87 | 7.62 | 11 | 22 | 0.00003 | 0.65 | 0.96 |
| Urology | 0.60 | 2.50 | 9 | 18 | 0.04671 | -0.17 | 0.89 |
| **8. Decision-making and problem-solving** |  |  |  |  |  |  |  |
| General surgery | 0.86 | 7.12 | 14 | 28 | 0.00001 | 0.67 | 0.95 |
| Gynecology | 0.79 | 4.77 | 16 | 48 | 0.00001 | 0.56 | 0.92 |
| Orthopedics | 0.79 | 4.82 | 15 | 15 | 0.00211 | 0.41 | 0.93 |
| Otorhinolaryngology | 0.68 | 3.14 | 11 | 22 | 0.01082 | 0.16 | 0.90 |
| Urology | 0.82 | 5.58 | 9 | 18 | 0.00097 | 0.48 | 0.95 |
| **9. Self-criticism and ability to learn from mistakes** |  |  |  |  |  |  |  |
| General surgery | 0.76 | 4.13 | 14 | 28 | 0.00069 | 0.43 | 0.91 |
| Gynecology | 0.75 | 4.01 | 16 | 48 | 0.00009 | 0.48 | 0.90 |
| Orthopedics | 0.76 | 4.21 | 15 | 15 | 0.00424 | 0.32 | 0.92 |
| Otorhinolaryngology | 0.62 | 2.60 | 11 | 22 | 0.02715 | -0.02 | 0.88 |
| Urology | 0.79 | 4.80 | 9 | 18 | 0.00229 | 0.39 | 0.94 |
| **10. Thoroughness** |  |  |  |  |  |  |  |
| General surgery | 0.78 | 4.58 | 14 | 28 | 0.00030 | 0.48 | 0.92 |
| Gynecology | 0.61 | 2.56 | 16 | 48 | 0.00609 | 0.18 | 0.84 |
| Orthopedics | 0.79 | 4.71 | 15 | 15 | 0.00237 | 0.39 | 0.93 |
| Otorhinolaryngology | 0.64 | 2.79 | 11 | 22 | 0.01938 | 0.05 | 0.89 |
| Urology | 0.80 | 5.12 | 9 | 18 | 0.00160 | 0.43 | 0.95 |
| **11. Organization and planning** |  |  |  |  |  |  |  |
| General surgery | 0.56 | 2.27 | 14 | 28 | 0.03171 | -0.05 | 0.84 |
| Gynecology | 0.70 | 3.33 | 16 | 48 | 0.00063 | 0.37 | 0.88 |
| Orthopedics | 0.81 | 5.18 | 15 | 15 | 0.00143 | 0.45 | 0.93 |
| Otorhinolaryngology | 0.71 | 3.50 | 11 | 22 | 0.00598 | 0.24 | 0.91 |
| Urology | 0.79 | 4.81 | 9 | 18 | 0.00226 | 0.39 | 0.94 |
| **12. Physical and mental endurance** |  |  |  |  |  |  |  |
| General surgery | 0.70 | 3.38 | 14 | 28 | 0.00295 | 0.30 | 0.89 |
| Gynecology | 0.67 | 3.01 | 16 | 48 | 0.00160 | 0.31 | 0.87 |
| Orthopedics | 0.67 | 3.07 | 15 | 15 | 0.01859 | 0.07 | 0.89 |
| Otorhinolaryngology | 0.81 | 5.18 | 11 | 22 | 0.00052 | 0.49 | 0.94 |
| Urology | 0.82 | 5.55 | 9 | 18 | 0.00100 | 0.47 | 0.95 |
| **13. Stress tolerance** |  |  |  |  |  |  |  |
| General surgery | 0.69 | 3.22 | 14 | 28 | 0.00406 | 0.26 | 0.89 |
| Gynecology | 0.69 | 3.23 | 16 | 48 | 0.00086 | 0.35 | 0.87 |
| Orthopedics | 0.68 | 3.13 | 15 | 15 | 0.01711 | 0.08 | 0.89 |
| Otorhinolaryngology | 0.68 | 3.12 | 11 | 22 | 0.01108 | 0.15 | 0.90 |
| Urology | 0.86 | 7.04 | 9 | 18 | 0.00024 | 0.58 | 0.96 |
| **14. Creativity and cognitive flexibility** |  |  |  |  |  |  |  |
| General surgery | 0.87 | 7.96 | 14 | 28 | 0.00000 | 0.70 | 0.95 |
| Gynecology | 0.82 | 5.67 | 16 | 48 | 0.00000 | 0.63 | 0.93 |
| Orthopedics | 0.82 | 5.52 | 15 | 15 | 0.00101 | 0.48 | 0.94 |
| Otorhinolaryngology | 0.68 | 3.15 | 11 | 22 | 0.01051 | 0.16 | 0.90 |
| Urology | 0.62 | 2.63 | 9 | 18 | 0.03867 | -0.11 | 0.90 |
| **15. Motivation** |  |  |  |  |  |  |  |
| General surgery | 0.77 | 4.31 | 14 | 28 | 0.00049 | 0.45 | 0.92 |
| Gynecology | 0.72 | 3.52 | 16 | 48 | 0.00037 | 0.41 | 0.89 |
| Orthopedics | 0.68 | 3.08 | 15 | 15 | 0.01828 | 0.07 | 0.89 |
| Otorhinolaryngology | 0.73 | 3.64 | 11 | 22 | 0.00481 | 0.27 | 0.91 |
| Urology | 0.93 | 15.33 | 9 | 18 | 0.00000 | 0.81 | 0.98 |
| **16. General assessment** |  |  |  |  |  |  |  |
| General surgery | 0.70 | 3.39 | 14 | 28 | 0.00292 | 0.30 | 0.89 |
| Gynecology | 0.73 | 3.69 | 16 | 48 | 0.00023 | 0.43 | 0.89 |
| Orthopedics | 0.84 | 6.42 | 15 | 15 | 0.00044 | 0.55 | 0.95 |
| Otorhinolaryngology | 0.68 | 3.12 | 11 | 22 | 0.01106 | 0.15 | 0.90 |
| Urology | 0.90 | 10.27 | 9 | 18 | 0.00002 | 0.71 | 0.97 |

**Table S5.** Correlations Between Scores on the Two Selection Tests and the Criteria, by Surgical Field (Phase 2).^a^

| **Dimension** | **n** | **VR technical aptitude test** | **GBA of cognitive abilities and personality** |
| --- | --- | --- | --- |
| **1. Medical knowledge** |  |  |  |
| General surgery | 16 | 0.07 (−0.46, 0.56) | 0.51 (−0.02, 0.83) |
| Gynecology | 17 | -0.33 (−0.69, 0.18) | 0.24 (−0.28, 0.64) |
| Orthopedics | 16 | 0.68 (0.26, 0.89) | 0.42 (−0.11, 0.78) |
| Otorhinolaryngology | 12 | 0.19 (−0.43, 0.70) | 0.4 (−0.21, 0.79) |
| Urology | 10 | 0.51 (−0.19, 0.87) | 0.49 (−0.22, 0.86) |
| **2. Technical skills** |  |  |  |
| General surgery | 16 | 0.62 (0.18, 0.87) | 0.35 (−0.20, 0.73) |
| Gynecology | 17 | 0.69 (0.32, 0.89) | 0.37 (−0.15, 0.74) |
| Orthopedics | 16 | 0.64 (0.21, 0.88) | 0.38 (−0.17, 0.74) |
| Otorhinolaryngology | 12 | 0.5 (−0.13, 0.84) | 0.32 (−0.32, 0.76) |
| Urology | 10 | 0.55 (−0.14, 0.88) | 0.07 (−0.61, 0.67) |
| **3. Communication with patients and their families** |  |  |  |
| General surgery | 16 | -0.28 (−0.68, 0.25) | 0.17 (−0.38, 0.63) |
| Gynecology | 17 | 0.19 (−0.34, 0.61) | 0.33 (−0.19, 0.69) |
| Orthopedics | 16 | 0.04 (−0.49, 0.55) | -0.01 (−0.52, 0.51) |
| Otorhinolaryngology | 12 | -0.18 (−0.69, 0.44) | 0.22 (−0.39, 0.72) |
| Urology | 10 | 0.62 (−0.04, 0.90) | 0.52 (−0.18, 0.88) |
| **4. Communication with medical staff and teamwork** |  |  |  |
| General surgery | 16 | -0.17 (−0.60, 0.38) | 0.25 (−0.30, 0.67) |
| Gynecology | 17 | 0.22 (−0.31, 0.64) | 0.64 (0.24, 0.87) |
| Orthopedics | 16 | 0.03 (−0.50, 0.55) | -0.14 (−0.58, 0.41) |
| Otorhinolaryngology | 12 | 0.09 (−0.54, 0.65) | -0.08 (−0.64, 0.53) |
| Urology | 10 | 0.63 (−0.02, 0.90) | 0.51 (−0.19, 0.87) |
| **5. Integrity** |  |  |  |
| General surgery | 16 | -0.18 (−0.61, 0.37) | 0.55 (0.09, 0.84) |
| Gynecology | 17 | 0.2 (−0.33, 0.63) | 0.45 (−0.07, 0.75) |
| Orthopedics | 16 | 0.36 (−0.18, 0.74) | 0.55 (0.09, 0.84) |
| Otorhinolaryngology | 12 | -0.24 (−0.72, 0.38) | -0.13 (−0.68, 0.50) |
| Urology | 10 | 0.52 (−0.18, 0.88) | 0.35 (−0.37, 0.82) |
| **6. Diligence** |  |  |  |
| General surgery | 16 | 0.05 (−0.48, 0.56) | 0.47 (−0.06, 0.81) |
| Gynecology | 17 | -0.12 (−0.57, 0.39) | 0.04 (−0.47, 0.52) |
| Orthopedics | 16 | 0.58 (0.12, 0.86) | 0.64 (0.21, 0.88) |
| Otorhinolaryngology | 12 | -0.36 (−0.79, 0.28) | 0.06 (−0.56, 0.63) |
| Urology | 10 | 0.54 (−0.15, 0.88) | 0.39 (−0.33, 0.83) |
| **7. Learning ability** |  |  |  |
| General surgery | 16 | -0.01 (−0.52, 0.50) | 0.43 (−0.11, 0.78) |
| Gynecology | 17 | -0.07 (−0.54, 0.42) | 0.34 (−0.18, 0.70) |
| Orthopedics | 16 | 0.5 (0.04, 0.83) | 0.36 (−0.18, 0.74) |
| Otorhinolaryngology | 12 | -0.22 (−0.72, 0.40) | 0.5 (−0.13, 0.84) |
| Urology | 10 | 0.65 (0.03, 0.91) | 0.45 (−0.26, 0.86) |
| **8. Decision-making and problem-solving** |  |  |  |
| General surgery | 16 | 0.25 (−0.30, 0.67) | 0.48 (−0.05, 0.81) |
| Gynecology | 17 | -0.14 (−0.59, 0.37) | 0.43 (−0.09, 0.74) |
| Orthopedics | 16 | 0.5 (0.04, 0.83) | 0.44 (−0.09, 0.79) |
| Otorhinolaryngology | 12 | 0.58 (−0.05, 0.86) | 0.38 (−0.24, 0.78) |
| Urology | 10 | 0.35 (−0.37, 0.82) | 0.21 (−0.52, 0.74) |
| **9. Self-criticism and ability to learn from mistakes** |  |  |  |
| General surgery | 16 | -0.03 (−0.54, 0.49) | 0.4 (−0.14, 0.76) |
| Gynecology | 17 | -0.06 (−0.53, 0.43) | 0.36 (−0.16, 0.7) |
| Orthopedics | 16 | 0.32 (−0.23, 0.72) | 0.23 (−0.32, 0.66) |
| Otorhinolaryngology | 12 | 0.1 (−0.52, 0.66) | 0.39 (−0.23, 0.78) |
| Urology | 10 | 0.6 (−0.07, 0.90) | 0.28 (−0.43, 0.79) |
| **10. Thoroughness** |  |  |  |
| General surgery | 16 | -0.13 (−0.57, 0.41) | 0.35 (−0.20, 0.73) |
| Gynecology | 17 | -0.34 (−0.70, 0.17) | -0.02 (−0.49, 0.46) |
| Orthopedics | 16 | 0.53 (0.07, 0.84) | 0.39 (−0.15, 0.76) |
| Otorhinolaryngology | 12 | -0.13 (−0.68, 0.50) | -0.25 (−0.72, 0.38) |
| Urology | 10 | 0.51 (−0.19, 0.87) | 0.26 (−0.45, 0.78) |
| **11. Organization and planning** |  |  |  |
| General surgery | 16 | -0.2 (−0.62, 0.36) | 0.47 (−0.06, 0.81) |
| Gynecology | 17 | -0.22 (−0.63, 0.30) | 0.11 (−0.41, 0) |
| Orthopedics | 16 | 0.29 (−0.26, 0.70) | 0.16 (−0.39, 0.63) |
| Otorhinolaryngology | 12 | -0.56 (−0.87, 0.07) | 0.14 (−0.49, 0.67) |
| Urology | 10 | 0.4 (−0.33, 0.83) | 0.36 (−0.36, 0.82) |
| **12. Physical and mental endurance** |  |  |  |
| General surgery | 16 | 0.08 (−0.45, 0.57) | 0.57 (0.10, 0.85) |
| Gynecology | 17 | 0.21 (−0.32, 0.63) | 0.61 (0.19, 0.86) |
| Orthopedics | 16 | 0.15 (−0.40, 0.61) | -0.08 (−0.57, 0.44) |
| Otorhinolaryngology | 12 | 0.3 (−0.33, 0.74) | -0.06 (−0.62, 0.55) |
| Urology | 10 | 0.59 (−0.09, 0.90) | 0.38 (−0.34, 0.83) |
| **13. Stress tolerance** |  |  |  |
| General surgery | 16 | 0.11 (−0.43, 0.59) | 0.48 (−0.05, 0.81) |
| Gynecology | 17 | 0.2 (−0.33, 0.63) | 0.62 (0.21, 0.87) |
| Orthopedics | 16 | 0.5 (0.04, 0.83) | 0.1 (−0.45, 0.59) |
| Otorhinolaryngology | 12 | 0.17 (−0.46, 0.69) | -0.08 (−0.64, 0.53) |
| Urology | 10 | 0.59 (−0.09, 0.90) | 0.38 (−0.34, 0.83) |
| **14. Creativity and cognitive flexibility** |  |  |  |
| General surgery | 16 | 0.31 (−0.24, 0.71) | 0.44 (−0.09, 0.79) |
| Gynecology | 17 | -0.19 (−0.61, 0.34) | 0.59 (0.16, 0.86) |
| Orthopedics | 16 | 0.34 (−0.21, 0.73) | 0.36 (−0.18, 0.74) |
| Otorhinolaryngology | 12 | 0.49 (−0.15, 0.84) | -0.36 (−0.79, 0.28) |
| Urology | 10 | 0.61 (−0.05, 0.90) | 0.57 (−0.12, 0.89) |
| **15. Motivation** |  |  |  |
| General surgery | 16 | -0.25 (−0.67, 0.30) | 0.44 (−0.09, 0.79) |
| Gynecology | 17 | 0.26 (−0.27, 0.68) | 0.56 (0.12, 0.84) |
| Orthopedics | 16 | 0.46 (−0.07, 0.80) | 0.11 (−0.44, 0.60) |
| Otorhinolaryngology | 12 | -0.19 (−0.69, 0.44) | 0.14 (−0.49, 0.67) |
| Urology | 10 | 0.44 (−0.28, 0.85) | 0.38 (−0.34, 0.83) |
| **16. General assessment** |  |  |  |
| General surgery | 16 | 0.03 (−0.50, 0.55) | 0.51 (−0.02, 0.83) |
| Gynecology | 17 | -0.11 (−0.57, 0.39) | 0.48 (−0.05, 0.80) |
| Orthopedics | 16 | 0.49 (0.03, 0.83) | 0.55 (0.09, 0.84) |
| Otorhinolaryngology | 12 | 0.12 (−0.51, 0.67) | 0.3 (−0.33, 0.74) |
| Urology | 10 | 0.54 (−0.15, 0.88) | 0.43 (−0.28, 0.85) |
| **17. Mean performance evaluation without technical skills** |  |  |  |
| General surgery | 16 | 0.03 (−0.50, 0.55) | 0.57 (0.10, 0.85) |
| Gynecology | 17 | 0.04 (−0.47, 0.52) | 0.59 (0.16, 0.86) |
| Orthopedics | 16 | 0.49 (0.03, 0.83) | 0.32 (−0.23, 0.72) |
| Otorhinolaryngology | 12 | 0.05 (−0.56, 0.63) | 0.24 (−0.37, 0.73) |
| Urology | 10 | 0.57 (−0.12, 0.89) | 0.4 (−0.33, 0.83) |

| **^a^** Values are Pearson correlations. 95% confidence intervals are presented in parentheses**.** |
| --- |

**Table S6.** Parameter Scores of Interns, Residents, and Expert Surgeons on the VR Technical Aptitude Test (ANOVA and Post Hoc Comparisons).

**One-way between-subjects ANOVA**

| **Parameter** | ***F*** | ***p*** | $\boldsymbol{\eta}^{\boldsymbol{2}}$ |
| --- | --- | --- | --- |
|  |  |  |  |
| **Success rate** | *F*(2, 211) = 33.91 | *p* < .001 | 0.24 |
| **Time** | *F*(2, 211) = 50.57 | *p* < .001 | 0.32 |
| **Number of mistakes** | *F*(2, 211) = 58.61 | *p* < .001 | 0.36 |
| **Path length** | *F*(2, 211) = 42.24 | *p* < .001 | 0.29 |
| **Percent of time within scope** | *F*(2, 211) = 11.69 | *p* < .001 | 0.10 |

**Post hoc comparisons using the Tukey HSD test**

| **Parameter** | ***Mean difference*  (z-scores)** | | **95% CI of difference** | | | | ***p*** | |
| --- | --- | --- | --- | --- | --- | --- | --- | --- |
|  |  |  | ***Lower*** | | ***Upper*** | |  |  |
| **Success rate** |  | |  | |  | |  | |
| Residents vs Interns | 0.33 | | 0.15 | | 0.50 | | *p* < .001 | |
| Experts vs Interns | 0.64 | | 0.46 | | 0.83 | | *p* < .001 | |
| Experts vs Residents | 0.32 | | 0.13 | | 0.50 | | *p* < .001 | |
| **Time** |  | |  | |  | |  | |
| Residents vs Interns | 0.45 | | 0.16 | | 0.75 | | *p* < .01 | |
| Experts vs Interns | 1.30 | | 0.98 | | 1.59 | | *p* < .001 | |
| Experts vs Residents | 0.84 | | 0.53 | | 1.14 | | *p* < .001 | |
| **Number of mistakes** |  | |  | |  | |  | |
| Residents vs Interns | 0.20 | | 0.03 | | 0.37 | | *p* < .05 | |
| Experts vs Interns | 0.80 | | 0.62 | | 0.98 | | *p* < .001 | |
| Experts vs Residents | 0.60 | | 0.42 | | 0.78 | | *p* < .001 | |
| **Path length** |  | |  | |  | |  | |
| Residents vs Interns | 0.26 | | 0.01 | | 0.51 | | *p* < .05 | |
| Experts vs Interns | 0.99 | | 0.73 | | 1.26 | | *p* < .001 | |
| Experts vs Residents | 0.74 | | 0.47 | | 1.00 | | *p* < .001 | |
| **Percent of time within scope** |  | |  | |  | |  | |
| Residents vs Interns | 0.08 | | -0.26 | | 0.42 | | ns | |
| Experts vs Interns | 0.67 | | 0.32 | | 1.02 | | *p* < .001 | |
| Experts vs Residents | 0.59 | | 0.23 | | 0.94 | | *p* < .001 | |
|  |  |  | |  | |  | |  |


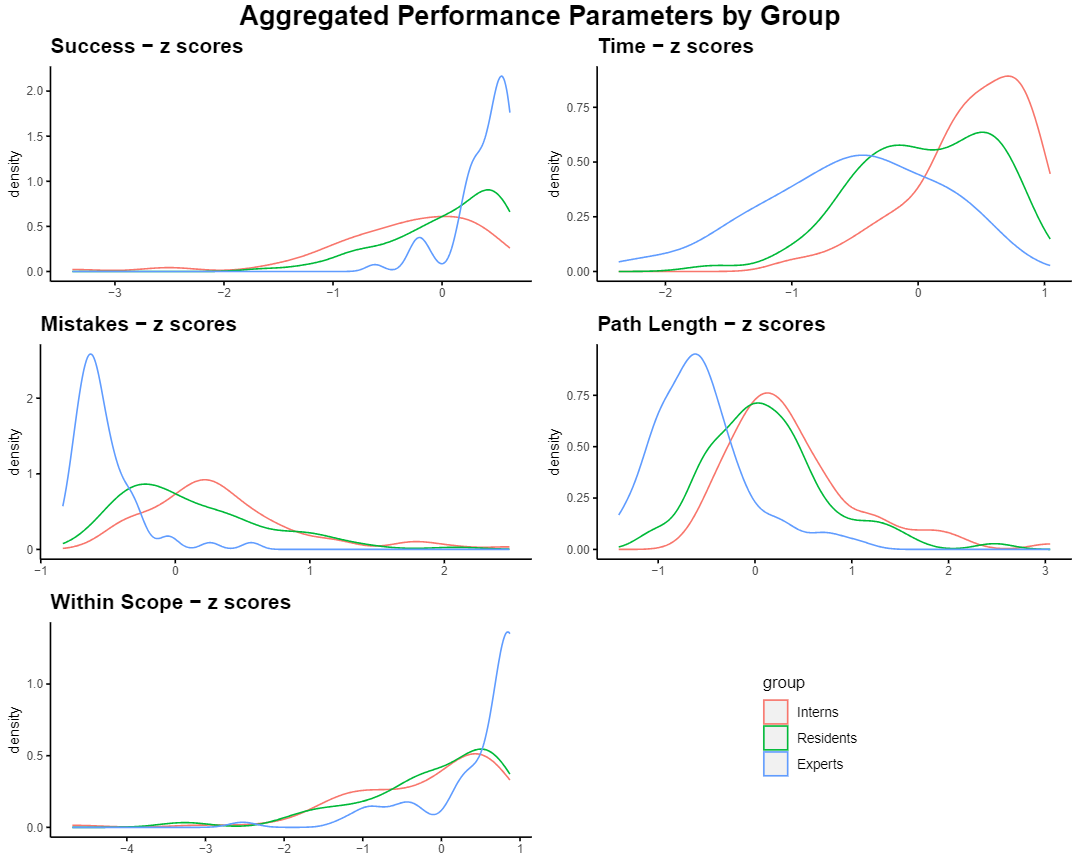
**Figure S1.** Distributions of Performance Parameters in the VR Technical Aptitude Test by Group.

**Table S7.** Summary of Multiple Regression Analyses for the Evaluation of Gender Bias.

**Multiple regression for evaluating gender bias in the technical aptitude test**

| ***Predictors*** | ***Estimates*** | ***Std. error*** | ***95% CI*** | ***t*** | ***p*** |
| --- | --- | --- | --- | --- | --- |
| **(Intercept)** | -0.01 | 0.13 | -0.27 – 0.24 | -0.17 | 0.915 |
| **Technical aptitude score** | 0.03 | 0.01 | 0.01 – 0.04 | 3.62 | 0.001 |
| **Gender [female]** | 0.06 | 0.24 | -0.42 – 0.53 | 0.25 | 0.805 |
| **Technical aptitude score x  Gender [female]** | 0.01 | 0.01 | -0.02 – 0.04 | 0.76 | 0.452 |
| **R^2^ / R^2^ adjusted** | 0.282 / 0.248 | | | |  |

**Multiple regression for evaluating gender bias in the GBA of cognitive abilities and personality characteristics**

| ***Predictors*** | ***Estimates*** | ***Std. error*** | ***95% CI*** | ***t*** | ***p*** |
| --- | --- | --- | --- | --- | --- |
| **(Intercept)** | 0.04 | 0.14 | -0.23 – 0.32 | 0.33 | 0.745 |
| **GBA score** | 0.02 | 0.01 | 0.01 – 0.04 | 2.70 | **0.009** |
| **Gender [female]** | -0.00 | 0.25 | -0.49 – 0.49 | -0.006 | 0.995 |
| **GBA score x  Gender [female]** | -0.01 | 0.01 | -0.03 – 0.02 | -0.44 | 0.659 |
| **R^2^ / R^2^ adjusted** | 0.140 / 0.097 | | | |  |

**Table S8.** Correlations Between Prior Simulator and Video Game Experience and Selection Test Scores (Phase 2).

| **Prior experience** | **VR technical aptitude test** | **GBA of cognitive abilities and personality** |
| --- | --- | --- |
| Simulator experience | 0.14 (−0.09, 0.37) | -0.01 (−0.24, 0.22) |
| Video game experience | 0.11 (−0.12, 0.34) | 0.16 (−0.08, 0.38) |

**Note**. None of the correlations reached statistical significance at *p* < .05 (two-tailed). Values are Pearson correlations. 95% confidence intervals are presented in parentheses**.**

**Table S9.** Summary of Multiple Regression Analyses for the Evaluation of Gender Bias (Sensitivity Analyses) .

**Multiple regression for evaluating gender bias in the technical aptitude test (adjusting for prior experience with simulators and video games)**

| ***Predictors*** | ***Estimates*** | ***Std. error*** | ***95% CI*** | ***t*** | ***p*** |
| --- | --- | --- | --- | --- | --- |
| **(Intercept)** | 0.04 | 0.35 | -0.66 – 0.74 | 0.903 | 0.04 |
| **Technical aptitude score** | 0.03 | 0.01 | 0.01 – 0.04 | 0.001 | 0.03 |
| **Gender [female]** | 0.04 | 0.26 | -0.49 – 0.56 | 0.893 | 0.04 |
| **Simulator experience** | 0.01 | 0.15 | -0.29 – 0.30 | 0.967 | 0.01 |
| **Video game experience** | -0.03 | 0.10 | -0.24 – 0.18 | 0.807 | -0.03 |
| **Technical aptitude score x  Gender [female]** | 0.01 | 0.02 | -0.02 – 0.04 | 0.473 | 0.01 |
| **R^2^ / R^2^ adjusted** | 0.285 / 0.252 | | | |  |

**Multiple regression for evaluating gender bias in the GBA of cognitive abilities and personality characteristics (adjusting for prior experience with video games)**

| ***Predictors*** | ***Estimates*** | ***Std. error*** | ***95% CI*** | ***t*** | ***p*** |
| --- | --- | --- | --- | --- | --- |
| **(Intercept)** | 0.22 | 0.32 | -0.42 – 0.85 | 0.500 | 0.22 |
| **GBA score** | 0.02 | 0.01 | 0.01 – 0.04 | 0.011 | 0.02 |
| **Gender [female]** | -0.07 | 0.27 | -0.60 – 0.47 | 0.809 | -0.07 |
| **Video game experience** | -0.07 | 0.11 | -0.29 – 0.16 | 0.553 | -0.07 |
| **GBA score x  Gender [female]** | -0.01 | 0.01 | -0.03 – 0.02 | 0.647 | -0.01 |
| **R^2^ / R^2^ adjusted** | 0.145 / 0.098 | | | |  |
